# Clinical examination findings were not associated with isometric strength or functional performance in adults with groin pain

**DOI:** 10.1186/s10195-026-00971-1

**Published:** 2026-10-15

**Authors:** Matheus Oliveira Branco, Tamiris Beppler Martins, Guilherme Vinicius da Costa, Vitor Mateus Madruga de Sousa, Alan Immich, Kaue Bertoto Dutra, Filippo Migliorini, Nicola Maffulli, Rodrigo Okubo

**Affiliations:** 1 Programa de Pós-Graduação em Fisioterapia, Universidade do Estado de Santa Catarina, Florianópolis, SC Brazil; 2 University Center Unifacvest, Lages, Santa Catarina Brazil; 3 Departamento de Fisioterapia, Centro de Ciências, Tecnologias e Saúde, Universidade Federal de Santa Catarina, Araranguá, SC Brazil; 4 La Trobe Sport and Exercise Medicine Research Centre, School of Allied Health, Human Services and Sport, La Trobe University, Melbourne, Australia; 5 University of Planalto Catarinense, Lages, Santa Catarina Brazil; 6 Department of Trauma and Reconstructive Surgery, University Hospital of Halle, Martin-Luther University Halle-Wittenberg, Ernst-Grube-Street 40, 06097 Halle, Saale Germany; 7 Department of Life Sciences, Health, and Health Professions, Link Campus University, Via del Casale di San Pio V, 00165 Rome, Italy; 8 Research Department of Clinical Orthopaedics, Eifelklinik St. Brigida, Simmerath, Germany; 9 Department of Medicine, Surgery and Dentistry, University of Salerno, 84081 Baronissi, Italy; 10 School of Pharmacy and Bioengineering, Keele University Faculty of Medicine, Stoke on Trent, ST4 7QB UK; 11 Centre for Sports and Exercise Medicine, Barts and the London School of Medicine and Dentistry, Mile End Hospital, Queen Mary University of London, London, E1 4DG UK

**Keywords:** Groin pain, Clinical examination, Hip muscle strength, Dynamic balance, Functional performance

## Abstract

**Objectives:**

The aim of this study was to investigate whether positive clinical examination findings were associated with selected measures of lower-limb isometric strength or functional performance in physically active adults with groin pain.

**Methods:**

This was a cross-sectional, observational and exploratory study with blinded outcome assessment. A total of 37 physically active adults (18–45 years) with clinically diagnosed groin pain (≥ 6 weeks) underwent standardised orthopaedic testing (flexion, abduction and external rotation [FABER]; flexion, adduction and internal rotation [FADIR]; scour; squeeze), targeted palpation (adductors, pubic symphysis), isometric hip and knee strength testing using a handheld dynamometer and functional performance tests (modified Star Excursion Balance Test [mSEBT], cross-over hop). Associations between clinical findings and strength/performance measures were analysed using adjusted binary logistic regression models controlling for age, body mass index and height, with false discovery rate correction.

**Results:**

Participants demonstrated broadly similar right- and left-side performance in the mSEBT and cross-over hop tests, with strength ratios close to 1.0. Positive clinical examination findings were common, particularly for left adductor-region palpation (74.3% of valid observations) and the left FABER test (70.3%). After adjustment for multiple comparisons, no significant associations were found between positive clinical tests and functional or strength measures.

**Conclusions:**

In this exploratory sample of physically active adults with groin pain, positive orthopaedic and palpation tests were not significantly associated with deficits in dynamic balance or isometric strength. These findings are limited to the selected isometric and general functional measures and reinforce the importance of a multi-modal assessment that incorporates clinical classification, eccentric adductor strength testing and sport-specific functional evaluations to guide rehabilitation and return-to-sport decisions.

**Supplementary information:**

The online version contains supplementary material available at https://doi.org/10.1186/s10195-026-00971-1.

## Introduction

Groin pain is a frequent and often debilitating complaint in professional and recreational athletes, resulting from multi-factorial causes including musculoskeletal, biomechanical and load-related factors [6, 21, 28]. It is a major cause of time loss, impacting performance and quality of life. Although most research focuses on elite athletes in soccer, rugby and hockey, its prevalence among recreationally active adults underscores the need for broader assessment and management strategies. The aetiology is commonly classified as adductor-related, hip joint pathology, inguinal related (sports hernia) or other causes [4, 8]. Adductor-related pain is most prevalent [21, 28], while labral tears and hernias are frequent contributors [6, 23]. Acetabular labrum tears are found in over 20% of athletes with groin pain, indicating underdiagnosis of intra-articular causes. Evidence supports multi-modal management combining exercise, particularly adductor strengthening, with load control and flexibility training [5, 13, 16, 29]. Preventive strategies such as preseason adductor strength monitoring can reduce injury incidence [10, 19]. Clinically, evaluation should integrate symptoms, orthopaedic examination, functional testing and objective strength assessment. Tests such as flexion, abduction and external rotation (FABER) and flexion, adduction and internal rotation (FADIR) identify hip pathology [4, 37], while palpation differentiates adductor pain [31]. Functional tests (modified Star Excursion Balance Test [mSEBT], hop tests) assess dynamic stability [25, 37], and isometric dynamometry quantifies hip and knee strength, with reduced adduction strength consistently reported [5, 18, 31]. However, the relationship between these findings remains unclear. Clarifying such associations could refine screening, integrate diagnostic and performance measures and guide rehabilitation [1, 11, 22]. Therefore, this study aimed to explore relationships between functional performance, lower-limb isometric strength and clinical examination findings in physically active adults with groin pain. Given limited evidence, no a priori hypotheses were formulated.

## Methods

### Study design

This was a cross-sectional, observational and exploratory study with blinded outcome assessment. The primary aim was to describe the physical performance, isometric strength and clinical examination findings in physically active adults with a clinical diagnosis of groin pain and to explore possible associations between these variables. The study adhered to the Declaration of Helsinki, and the protocol was approved by the Research Ethics Committee of the Santa Catarina State University (CAAE 42853720.0.0000.0118, approval number 4.631.512). All participants provided written informed consent before participation.

### Eligibility criteria

Participants were recruited between March and August 2023 through digital advertisements, local training centres, sports clubs, rehabilitation clinics and university channels. Eligible participants were required to be between 18 and 45 years old, have a clinical diagnosis of groin pain confirmed by a physiotherapist or sports medicine physician and have symptoms persisting for at least 6 weeks. They also had to be classified as ‘moderately active’ or ‘very active’ according to the World Health Organisation guidelines [2], which require engaging in more than 150 min of physical activity per week. Another criterion was performing lower-limb training at least twice per week over the past 6 months. Exclusion criteria comprised pain exclusively referred to the lumbar spine, history of surgery in the lumbar spine, pelvis or lower limbs and history of fractures in the pelvis or lower limbs. Participants with diagnosed abdominal or inguinal hernias, neurological symptoms in the lower limbs, severe muscle weakness or deconditioning or neuromusculoskeletal conditions that could compromise performance or assessment were also excluded. In total, 37 participants met the eligibility criteria and were included in the analysis. Eligibility was not restricted by symptom laterality.

### Data collection procedures

All assessments were performed in a single session in the biomechanics laboratory under controlled ambient conditions (temperature 22–24 °C, humidity 45–55%). Participants refrained from vigorous exercise for 24 h prior to testing and wore athletic clothing and sports shoes of their choice. The study included a structured questionnaire covering demographic information and activity-related information, a physical activity profile (type, weekly volume and frequency), a history of groin pain and other musculoskeletal complaints and strategies for injury prevention. Anthropometric measures were also obtained: height was measured using a fixed wall-mounted stadiometer with 0.1 cm accuracy; body mass was assessed with a calibrated digital scale accurate to 0.1 kg; body mass index (BMI) was calculated as kg/m^2^; and leg length was measured in the supine position, from the anterior superior iliac spine to the distal edge of the medial malleolus, using a flexible tape with 0.1 cm accuracy. For the present analysis, age and anthropometric measures were available in the de-identified analytical dataset; sex and participant-level symptom laterality were not retained. Side-specific physical performance, strength and clinical examination variables were retained according to anatomical side (right/left) and were not recoded as symptomatic versus asymptomatic side.

### Functional performance tests

**Fig. 1:**
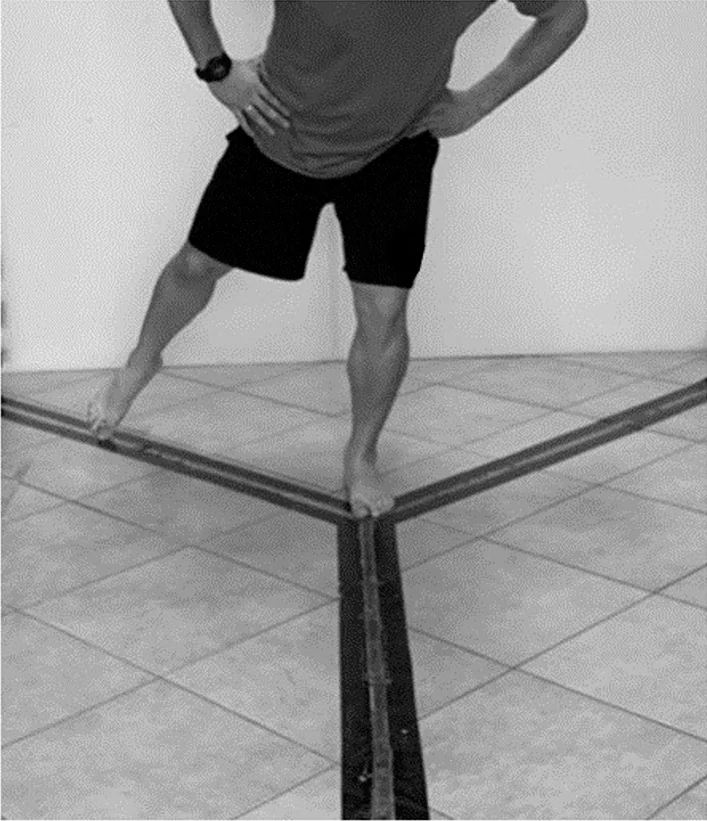
Functional performance testing. Modified Star Excursion Balance Test (mSEBT), which is performed in three directions (anterior, posteromedial and posterolateral). The image illustrates the posterolateral (PL) reach

The mSEBT was conducted in three directions: anterior (A), posteromedial (PM) and posterolateral (PL) (Fig. 1) [9]. Participants performed the test barefoot, standing with the stance foot centred on the grid intersection and hands placed on the hips. They were instructed to reach maximally in the target direction with the contralateral limb, lightly touching the ground with the great toe without transferring weight. Trials were considered invalid if the stance foot moved, weight was shifted onto the reaching foot or if the hands left the hips. Each participant completed three practice trials in each direction, followed by three valid recorded trials. Reach distances were normalised to leg length and expressed as percentages, and the mean of the three recorded trials was used for analysis.

**Fig. 2:**
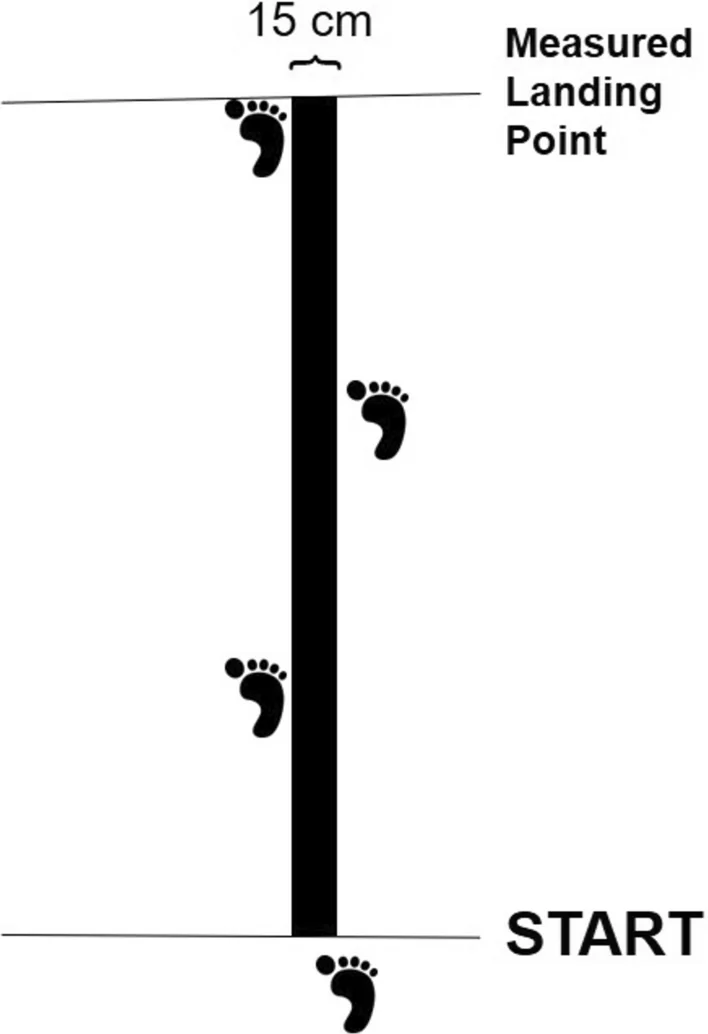
Cross-over hop test. The participant performs three consecutive single-leg hops crossing over a central 15 cm line. The total distance is measured from the starting point to the final measured landing point

The cross-over hop test was performed according to the protocol described by Reid et al. [26]. Participants began standing on one leg positioned behind the centre of a taped cross on the floor. They were instructed to hop forward and laterally, crossing the centre each time, for a total of three consecutive hops (Fig. 2). The test outcome was determined by measuring the distance in centimetres from the starting point to the final landing point. Each participant completed two practice trials, followed by two recorded trials, with the best result considered for analysis.

### Isometric strength testing

Isometric strength was measured using a calibrated portable dynamometer, HHD MEDEOR^®^ (MEDEOR^®^SP Tech; Santa Catarina, Brazil), with non-elastic straps for stabilisation. This method has shown excellent reliability for large lower limb muscle groups, with intraclass correlation coefficients (ICC) ranging from 0.85 to 0.99 (95% CI: 0.74–0.99) across quadriceps, hamstrings, hip abductors/adductors and rotators [3, 17, 33]. This approach was selected because it is feasible in clinical settings, demonstrates good reliability and is commonly used in rehabilitation practice.

**Fig 3.**
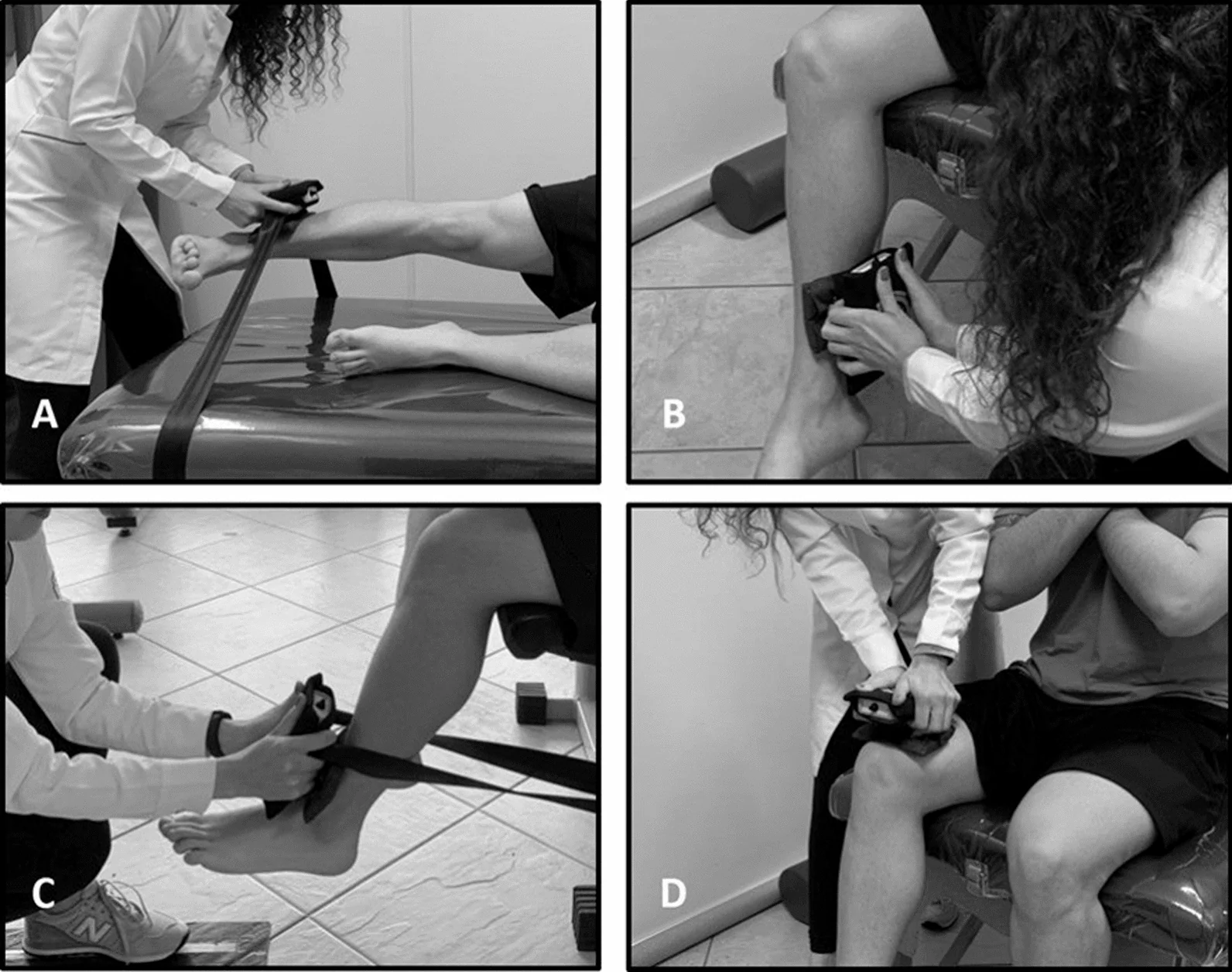
Isometric strength testing using a handheld dynamometer (HHD). **A** Hip abduction with belt fixation, **B** hip internal/external rotation, **C** knee extension with belt and **D** hip flexion

The testing sequence included the following assessments (Fig. 3): hip abduction and adduction were tested in lateral decubitus with the hip in a neutral position and the knee extended, with the dynamometer placed on the lateral or medial malleolus (ICC 0.88–0.95) (Fig. 3A); hip internal and external rotation were tested in a seated position with the hip and knee flexed at 90°, with the dynamometer positioned on the medial or lateral aspect of the distal leg (ICC 0.82–0.93) (Fig. 3B); knee extension was tested in a seated position with the hip at 90° and the knee flexed at 60°, with the dynamometer placed 5 cm proximal to the lateral malleolus (ICC 0.89–0.99) (Fig. 3C); knee flexion was tested in the prone position with the hip in neutral and the knee flexed at 60°, with the dynamometer fixed distally on the leg near the malleolus and stabilised using a non-elastic strap (ICC 0.85–0.97); and hip flexion was tested in a seated position with the hip at 90° and the knee flexed, with the dynamometer placed on the anterior thigh proximal to the knee (ICC 0.87–0.94) (Fig. 3D).

Each contraction lasted 5 s with 30 s rest between attempts. Three maximal efforts were performed per movement, preceded by a warm-up trial. Peak force (kgf) was recorded, and the mean of the three trials was used. Strength ratios were calculated as follows: the flexion-to-extension ratio (F/E) was obtained by dividing knee flexion by knee extension strength; the adduction-to-abduction ratio (Add/Abd) was calculated as hip adduction divided by hip abduction strength; and the internal-to-external rotation ratio (IR/ER) was determined by dividing hip internal rotation by hip external rotation strength. Absolute peak force was reported in kgf because the handheld dynamometer directly provided force in this unit and the protocol was designed around a clinically feasible dynamometry procedure. Body-mass-normalised force and torque-based measures were not available for the present dataset.

### Clinical examination findings

Clinical examination findings were assessed bilaterally by a physiotherapist (> 5 years’ experience), who was blinded to other measurements. Reliability of the included orthopaedic and palpation tests varies from moderate to good, with ICC values typically between 0.60 and 0.85, depending on the manoeuvrer [27, 34].

**Fig. 4:**
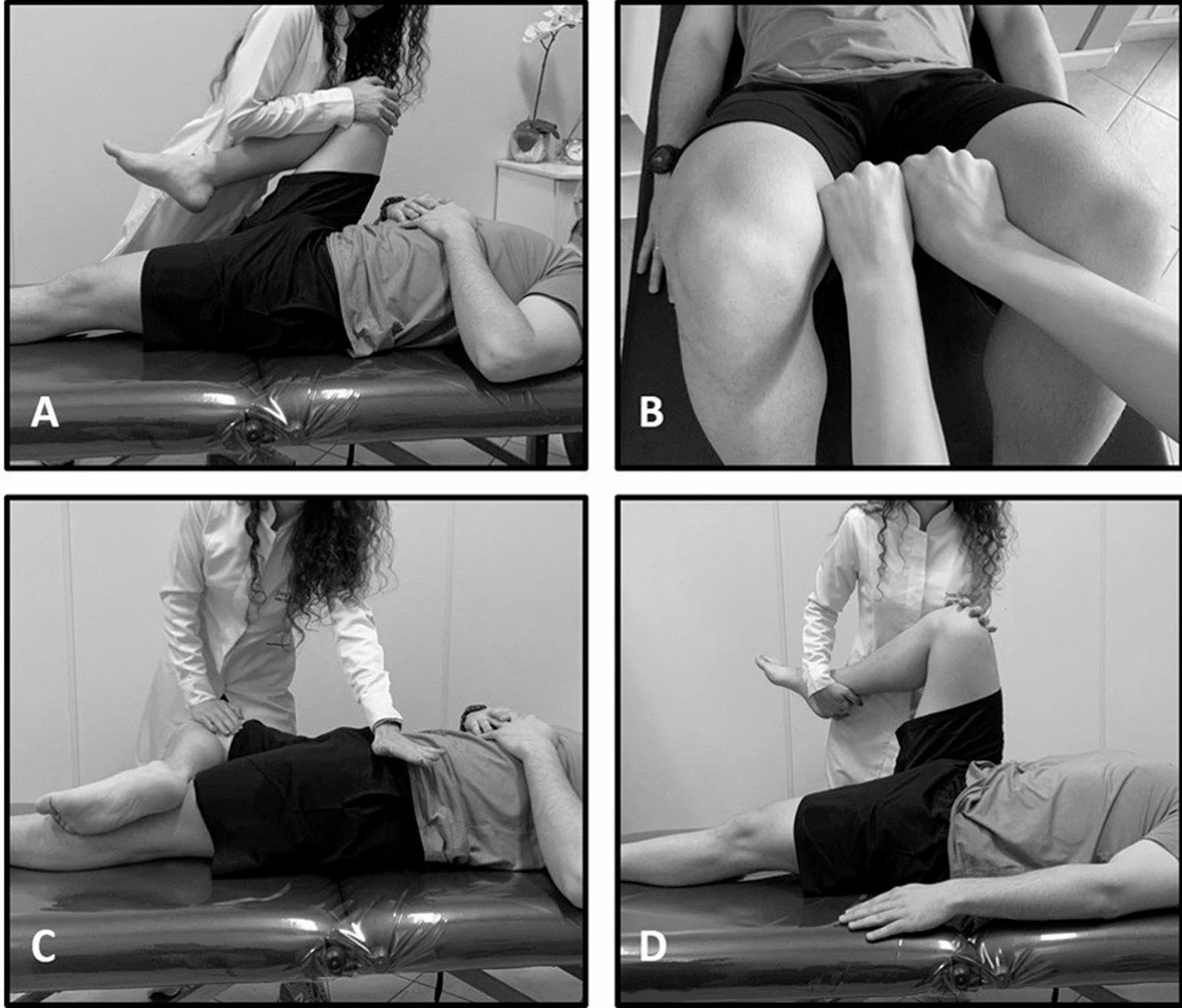
Clinical examination findings: orthopaedic tests and palpation procedures. **A** Hip scour test, **B** adductor squeeze test at 45 degrees, **C** FABER test (flexion, abduction, external rotation) and **D** FADIR test (flexion, adduction, internal rotation)

The testing sequence included the following assessments (Fig. 4): the hip scour test was performed with the hip passively flexed and adducted under axial load, followed by movement into abduction (ICC 0.61–0.77) (Fig. 4A); the adductor squeeze test was conducted at 0°, 45° and 90° of hip flexion using a dynamometer or the examiner’s fist positioned between the participant’s knees, who was instructed to squeeze maximally for 5 s while pain reproduction was recorded (ICC 0.89–0.97) (Fig. 4B); the FABER test involved hip flexion, abduction and external rotation with the foot placed on the opposite thigh (ICC 0.67–0.85) (Fig. 4C); the FADIR test was performed with the hip flexed at 90°, adducted and internally rotated (ICC 0.50–0.71) (Fig. 4D); and palpation was carried out over the adductor origin, pubic symphysis, quadriceps belly and lower abdominal region (ICC 0.79–0.91 for adductor palpation). A positive finding in all tests was defined as the reproduction of the participant’s typical pain.

### Statistical analysis

All analyses were performed using IBM SPSS Statistics for Windows, version 20.0 (IBM Corp.; Armonk, NY, USA). Descriptive statistics were used to summarise the sample characteristics. Continuous variables were expressed as mean and standard deviation (SD) when normally distributed or as median and interquartile range (IQR) when the Shapiro–Wilk test indicated non-normality. Categorical variables were presented as absolute (*n*) and relative (%) frequencies. Associations between functional performance or isometric strength variables and the presence of positive clinical examination findings (orthopaedic manoeuvrers, palpation sites and squeeze positions) were assessed using binary logistic regression models adjusted for age, body mass index (BMI) and height. For each clinical outcome, all relevant predictors were entered into separate models, and odds ratios (ORs) with 95% confidence intervals (95% CIs) were reported. The Benjamini–Hochberg false discovery rate (FDR) correction was applied to control for multiple comparisons within each outcome. Statistical significance was set at *p* < 0.05 for all analyses. Given the exploratory design and sample size, no a priori power calculation was performed, and the analyses were interpreted as estimates of association rather than confirmatory tests. Side-specific variables were analysed by anatomical side (right/left) rather than by symptomatic versus asymptomatic side because participant-level symptom laterality was unavailable.

## Results

### Anthropometric characteristics

The study included 37 physically active adults diagnosed with groin pain. Sex and participant-level symptom laterality (right-sided, left-sided or bilateral) were not retained in the de-identified analytical dataset, and therefore their distributions could not be reconstructed for the present analysis. Table 1 summarises the anthropometric data. Mean age was 30.19 ± 7.41 years, with an average BMI in the overweight range (27.21 ± 3.95 kg/m^2^). Mean leg length was 99.97 ± 4.07 cm.

**Table 1:** Anthropometric characteristics

Variable	Values^*X* ± SD [95% CI]^	Units
Age	30.19 ± 7.41 [27.72–32.66]	Years
Body mass	86.69 ± 15.63 [81.47–91.90]	kg
Height	1.78 ± 0.06 [1.76–1.80]	m
BMI	27.21 ± 3.95 [25.90–28.53]	kg/m^2^
Leg length	99.97 ± 4.07 [98.62–101.33]	cm

*X*, mean; SD, standard deviation; CI, confidence interval; kg, kilogram; m, metre; cm, centimetre

### Functional performance tests

Participants demonstrated broadly similar right- and left-side performance in the mSEBT across directions. Cross-over hop distances were also similar between anatomical sides (Table 2). These comparisons were not recoded as symptomatic versus asymptomatic side.

**Table 2:** Functional tests (mSEBT and cross-over hop test)

Variables^*X* ± SD [95% CI]^		Values	Units
mSEBT A	Right Left	54.73 ± 15.12 [49.69–59.77] 56.41 ± 11.69 [52.51–60.30]	% %
mSEBT_PM	Right Left	81.95 ± 12.85 [77.66–86.23] 74.59 ± 28.85 [64.98–84.21]	% %
mSEBT_PL	Right Left	86.30 ± 27.41 [77.16–95.44] 92.89 ± 8.05 [90.21–95.58]	% %
Cross-over hop	Right Left	286.22 ± 62.88 [265.25–307.18] 280.97 ± 57.62 [261.76–300.18]	cm cm

*X*, mean; SD, standard deviation; CI, confidence interval; cm, centimetre; mSEBT, modified Star Excursion Balance Test; A, anterior direction; PL, posterolateral direction; PM, posteromedial direction, %, percentage of leg length

### Muscle strength and ratios

Maximal isometric strength values and muscle balance ratios are presented in Table 3. Flexion/extension, adduction/abduction and internal/external rotation ratios were close to 1.0.

**Table 3:** Muscle strength and ratios

Variables^*X* ± SD [95% CI]^		Values	Units
Peak knee flexion strength	Right Left	30.15 ± 7.42 [27.67–32.62] 29.52 ± 5.71 [27.62–31.43]	kgf kgf
Peak knee extension strength	Right Left	50.88 ± 13.59 [46.35–55.41] 47.14 ± 11.39 [43.35–50.94]	kgf kgf
F/E ratio	Right Left	0.624 ± 0.183 [0.563–0.685] 0.653 ± 0.163 [0.598–0.707]	
Peak hip abduction strength	Right Left	15.93 ± 3.37 [14.80–17.05] 14.78 ± 3.00 [13.79–15.78]	kgf kgf
Peak hip adduction strength	Right Left	15.40 ± 4.70 [13.84–16.97] 16.45 ± 4.67 [14.89–18.01]	kgf kgf
Add/Abd ratio	Right Left	0.994 ± 0.337 [0.881–1.106] 1.133 ± 0.341 [1.020–1.247]	
Peak hip ER strength	Right Left	15.23 ± 4.83 [13.62–16.84] 14.78 ± 4.42 [13.31–16.26]	kgf kgf
Peak hip IR strength	Right Left	17.56 ± 5.01 [15.89–19.24] 19.44 ± 6.18 [17.38–21.50]	kgf kgf
IR/ER ratio	Right Left	1.204 ± 0.328 [1.094–1.313] 1.357 ± 0.375 [1.232–1.482]	
Peak hip flexion strength	Right Left	22.37 ± 4.35 [20.92–23.83] 22.38 ± 4.55 [20.87–23.90]	kgf kgf

*X*, mean; SD, standard deviation; CI, confidence interval; kgf, kilogram-force; F/E, flexion/extension; Add/Abd, adduction/abduction; IR, internal rotation; ER, external rotation

### Clinical examination findings (orthopaedic tests, palpation and squeeze test)

Positive clinical examination findings were frequent across several sites and manoeuvrers. The highest positivity rates among valid observations were for left adductor-region palpation (26/35, 74.3%) and the left FABER test (26/37, 70.3%) (Table 4).

**Table 4:** Clinical test frequencies

Clinical test		Positive (%)
Hip scour test	Right Left	13/37 (35.1) 23/37 (62.2)
FADIR test	Right Left	19/37 (51.4) 22/37 (59.5)
FABER test	Right Left	21/37 (56.8) 26/37 (70.3)
Squeeze at 90°		9/37 (24.3)
Squeeze at 45°		8/37 (21.6)
Squeeze at 0°		11/37 (29.7)
Palpation – adductor region	Right Left	24/37 (64.9) 26/35 (74.3)
Palpation – pubic symphysis		17/36 (47.2)
Palpation – quadriceps region	Right Left	17/37 (45.9) 23/37 (62.2)
Palpation – lower abdomen region	Right Left	16/37 (43.2) 22/37 (59.5)

Values are presented as positive cases/valid observations (%). Two observations were missing for left adductor-region palpation, and one observation was missing for pubic symphysis palpation

### Associations between functional/strength predictors and clinical outcomes

Logistic regression models adjusted for age, BMI and height assessed associations between functional performance or isometric strength variables and positive clinical examination findings. None of the 448 outcome–predictor models remained statistically significant after Benjamini–Hochberg FDR correction (all FDR-adjusted *p*-values ≥ 0.270). The smallest adjusted *p*-values were observed for the squeeze test at 45° with peak right hip adduction strength (OR 0.713, 95% CI 0.537–0.946; raw *p* = 0.019; FDR-adjusted *p* = 0.270) and peak left hip external rotation strength (OR 0.529, 95% CI 0.316–0.885; raw *p* = 0.015; FDR-adjusted *p* = 0.270). Complete model results, including positive and negative case counts, ORs, 95% CIs, raw *p*-values and FDR-adjusted *p*-values, are reported in Supplementary Table S1.

## Discussion

The most important findings of this study were that, despite frequent positive clinical examination findings, physically active adults with groin pain showed broadly similar right- and left-side values for isometric strength and functional performance, and none of the 448 adjusted outcome–predictor models remained statistically significant after FDR correction. The aim of this study was to explore whether clinical examination findings were associated with lower-limb isometric strength and generic functional performance in this cohort. Taken together, the results suggest that positive orthopaedic or palpation tests may reflect pain provocation or local tissue irritability without necessarily corresponding to measurable deficits in the specific isometric and functional measures used here. These comparisons were based on anatomical side and should not be interpreted as symptomatic-versus-asymptomatic comparisons.

These findings are consistent with the literature, which shows that clinical pain-provocation tests and objective performance measures do not always overlap in patients with groin pain. Positive left adductor-region palpation and a positive left FABER test were common in our sample, while positive findings were also distributed across pubic and lower abdominal sites; this is compatible with the heterogeneous presentation of groin pain across adductor-, pubic- and inguinal-related entities [4, 7, 30]. Likewise, FABER, FADIR and related manoeuvrers may reproduce symptoms linked to local joint or periarticular irritability, but they do not necessarily identify reduced muscle performance or balance deficits [15, 24, 27, 34, 36]. This helps explain why clinical examination findings were frequent even when mSEBT, cross-over hop and isometric strength values were broadly similar between right and left sides. Importantly, anatomical side-to-side similarity does not establish affected-versus-unaffected symmetry because participant-level symptomatic-side classification was unavailable.

The lack of significant associations should also be interpreted in light of the selected outcome measures. Isometric dynamometry and generic functional tests were chosen because they are feasible, clinically accessible and supported by good reliability [3, 17, 33]. However, groin pain, particularly adductor-related presentations, may be more strongly linked to eccentric capacity, rate of force development, endurance or sport-specific load tolerance than to static strength or generic balance performance [21, 31, 32]. Previous studies have shown that eccentric adductor deficits can be present even when isometric measures appear preserved [31], while broad functional tasks may fail to discriminate symptomatic from asymptomatic athletes [14, 20]. Accordingly, our results should be interpreted as applying specifically to isometric strength and the functional tests included in this study rather than to muscle strength or functional capacity more broadly. The use of absolute force values should also be considered. Strength was reported in kgf as directly recorded by the handheld dynamometer to preserve clinical interpretability; however, absolute force may be influenced by body size, sex and lever-arm length. Although BMI and height were included as covariates, body-mass-normalised and torque-based measures were unavailable. Sex was not retained in the analytical dataset, precluding sex-specific analyses and preventing direct assessment of its influence on absolute force values.

Another important point is that the present study was exploratory and not powered for definitive inference. Several nominal associations had raw *p*-values below 0.05, but none remained statistically significant after FDR adjustment. The smallest adjusted *p*-values were 0.270; therefore, the observed estimates should be interpreted as imprecise, hypothesis-generating signals rather than confirmatory associations. Small but potentially clinically relevant relationships may have gone undetected given the modest sample size and the number of tested models.

From a clinical perspective, the results reinforce the importance of multi-modal assessment. The distribution of positive findings across pubic, abdominal and adductor-related sites suggests that not all participants represented the same clinical entity under the Doha framework [4, 8]. Because different groin pain subtypes may present with distinct impairments, clinical examination findings should be interpreted alongside symptom classification, targeted strength testing, especially eccentric adductor assessment, patient-reported symptoms and sport-specific functional demands [4, 7, 12, 31, 35]. This integrated approach is likely more useful for rehabilitation planning and return-to-sport decisions than relying on positive pain-provocation tests in isolation.

This study has several limitations. First, the modest sample size limited statistical power and increased the risk of type II error. Accordingly, non-significant findings should be interpreted with caution, as smaller but potentially clinically relevant associations may have gone undetected. Given the exploratory design, the present analyses should be considered hypothesis-generating rather than confirmatory, with interpretation focusing on the magnitude and direction of observed effects and their confidence intervals rather than on statistical significance alone. In this context, participants were analysed as a single cohort despite the recognised heterogeneity of groin pain presentations, including adductor-, pubic- and abdominal-related involvement. Although clinical findings were interpreted within the framework of the Doha Agreement, no formal subgroup analyses were performed, as the available sample size would not have supported adequately powered stratified analyses. Consequently, the generalisability of the findings across specific groin pain entities should be interpreted with caution. In addition, the scope of the present findings is limited by the selection of outcome measures. Isometric strength testing and general functional tests were chosen for their feasibility, reliability and common use in clinical practice; however, these measures do not capture eccentric strength, endurance or sport-specific load tolerance, which are particularly relevant in groin pain. Consequently, the present findings are limited to isometric strength and general functional performance and should not be extrapolated to broader measures of muscle strength or functional capacity. Participant-level symptom laterality was not retained in the analytical dataset, so right–left analyses could not be recoded as symptomatic versus asymptomatic side; consequently, right–left similarity cannot be interpreted as affected–unaffected symmetry. Absolute strength was reported in kgf, and body-mass-normalised or torque-based measures were not available. Absolute force can be influenced by body size, sex and lever-arm length; although BMI and height were included as covariates, residual influence of these factors cannot be excluded. Sex was also unavailable in the analytical dataset, preventing sex-specific analyses.

In practice, positive orthopaedic and palpation tests should be interpreted as part of a broader assessment rather than as stand-alone proxies of strength or functional impairment. Integrating clinical classification, eccentric and endurance testing and sport-specific evaluation may better guide individualised rehabilitation and return-to-sport decisions.

## Conclusions

In this exploratory sample of physically active adults with groin pain, positive orthopaedic and palpation tests were not significantly associated with deficits in dynamic balance or isometric lower-limb strength. These findings are limited to the selected isometric and generic functional measures and should be interpreted cautiously, given the sample size and heterogeneity of clinical presentations. Multi-modal evaluation, including clinical classification, targeted eccentric/endurance testing and sport-specific assessment, may better inform rehabilitation and return-to-sport decisions.

## Supplementary information

Supplementary Material 1.

## Data Availability

Data are available upon reasonable request from the corresponding author.

## Declarations

### Patient involvement

Study participants were not involved in the design, conduct, interpretation or translation of the current research.
