# Supplementary material for: Clinical examination findings were not associated with isometric strength or functional performance in adults with groin pain
Source: J Orthop Traumatol. 2026 Oct 15;27:60. doi: 10.1186/s10195-026-00971-1 (PMC13672736; doi:10.1186/s10195-026-00971-1)
Supplement: Supplementary file 1 [file 10195_2026_971_MOESM1_ESM.docx]

**Supplementary Table S1. Complete adjusted binary logistic regression results**

Separate binary logistic regression models were fitted for each clinical outcome-predictor combination and adjusted for age, body mass index (BMI), and height. Benjamini-Hochberg false discovery rate (FDR) correction was applied within each clinical outcome. Outcome values outside the prespecified binary coding (1 = positive, 2 = negative) were treated as missing, and models were fitted using available complete cases.

| **Clinical outcome** | **Predictor** | **Positive cases (n)** | **Negative cases (n)** | **OR** | **95% CI** | **Raw p value** | **FDR-adjusted p value** |
| --- | --- | --- | --- | --- | --- | --- | --- |
| **Hip Scour test R** | Peak Knee Flexion Strength R | 13 | 24 | 1.044 | 0.945-1.154 | 0.394 | 0.890 |
| Hip Scour test R | Peak Knee Flexion Strength L | 13 | 24 | 1.022 | 0.889-1.174 | 0.763 | 0.890 |
| Hip Scour test R | Peak Knee Extension Strength R | 13 | 24 | 0.978 | 0.923-1.036 | 0.449 | 0.890 |
| Hip Scour test R | Peak Knee Extension Strength L | 13 | 24 | 0.983 | 0.916-1.055 | 0.636 | 0.890 |
| Hip Scour test R | F/E Ratio R | 13 | 24 | 29.924 | 0.476-1882.704 | 0.108 | 0.890 |
| Hip Scour test R | F/E Ratio L | 13 | 24 | 6.979 | 0.085-573.722 | 0.388 | 0.890 |
| Hip Scour test R | Peak Hip Abduction Strength R | 13 | 24 | 0.868 | 0.671-1.122 | 0.279 | 0.890 |
| Hip Scour test R | Peak Hip Abduction Strength L | 13 | 24 | 0.872 | 0.656-1.159 | 0.344 | 0.890 |
| Hip Scour test R | Peak Hip Adduction Strength R | 13 | 24 | 1.002 | 0.863-1.163 | 0.979 | 0.994 |
| Hip Scour test R | Peak Hip Adduction Strength L | 13 | 24 | 0.948 | 0.807-1.115 | 0.521 | 0.890 |
| Hip Scour test R | Add/Abd Ratio R | 13 | 24 | 2.399 | 0.281-20.506 | 0.424 | 0.890 |
| Hip Scour test R | Add/Abd Ratio L | 13 | 24 | 1.008 | 0.124-8.202 | 0.994 | 0.994 |
| Hip Scour test R | Peak Hip External Rotation Strength R | 13 | 24 | 0.930 | 0.795-1.089 | 0.369 | 0.890 |
| Hip Scour test R | Peak Hip External Rotation Strength L | 13 | 24 | 0.942 | 0.793-1.119 | 0.498 | 0.890 |
| Hip Scour test R | Peak Hip Internal Rotation Strength R | 13 | 24 | 0.947 | 0.812-1.105 | 0.491 | 0.890 |
| Hip Scour test R | Peak Hip Internal Rotation Strength L | 13 | 24 | 0.974 | 0.863-1.099 | 0.666 | 0.890 |
| Hip Scour test R | IR/ER Ratio R | 13 | 24 | 1.201 | 0.139-10.375 | 0.868 | 0.935 |
| Hip Scour test R | IR/ER Ratio L | 13 | 24 | 1.280 | 0.200-8.214 | 0.794 | 0.890 |
| Hip Scour test R | Peak Hip Flexion Strength R | 13 | 24 | 1.122 | 0.919-1.370 | 0.259 | 0.890 |
| Hip Scour test R | Peak Hip Flexion Strength L | 13 | 24 | 1.026 | 0.873-1.205 | 0.759 | 0.890 |
| Hip Scour test R | mSEBT_A R | 13 | 24 | 1.032 | 0.961-1.109 | 0.385 | 0.890 |
| Hip Scour test R | mSEBT_A L | 13 | 24 | 0.961 | 0.892-1.035 | 0.296 | 0.890 |
| Hip Scour test R | mSEBT_PM R | 13 | 24 | 1.013 | 0.955-1.073 | 0.672 | 0.890 |
| Hip Scour test R | mSEBT_PM L | 13 | 24 | 1.026 | 0.987-1.066 | 0.194 | 0.890 |
| Hip Scour test R | mSEBT_PL R | 13 | 24 | 0.992 | 0.968-1.018 | 0.556 | 0.890 |
| Hip Scour test R | mSEBT_PL L | 13 | 24 | 0.982 | 0.897-1.076 | 0.701 | 0.890 |
| Hip Scour test R | Cross-Over Hop R | 13 | 24 | 1.002 | 0.990-1.014 | 0.723 | 0.890 |
| Hip Scour test R | Cross-Over Hop L | 13 | 24 | 0.998 | 0.985-1.011 | 0.770 | 0.890 |
| **Hip Scour test L** | Peak Knee Flexion Strength R | 23 | 14 | 1.034 | 0.928-1.152 | 0.546 | 0.728 |
| Hip Scour test L | Peak Knee Flexion Strength L | 23 | 14 | 0.983 | 0.855-1.130 | 0.808 | 0.970 |
| Hip Scour test L | Peak Knee Extension Strength R | 23 | 14 | 0.975 | 0.921-1.032 | 0.377 | 0.711 |
| Hip Scour test L | Peak Knee Extension Strength L | 23 | 14 | 0.950 | 0.882-1.023 | 0.172 | 0.687 |
| Hip Scour test L | F/E Ratio R | 23 | 14 | 3.995 | 0.066-240.632 | 0.508 | 0.711 |
| Hip Scour test L | F/E Ratio L | 23 | 14 | 7.464 | 0.069-808.606 | 0.400 | 0.711 |
| Hip Scour test L | Peak Hip Abduction Strength R | 23 | 14 | 0.834 | 0.644-1.079 | 0.167 | 0.687 |
| Hip Scour test L | Peak Hip Abduction Strength L | 23 | 14 | 0.644 | 0.435-0.954 | 0.028 | 0.394 |
| Hip Scour test L | Peak Hip Adduction Strength R | 23 | 14 | 1.001 | 0.864-1.160 | 0.992 | 0.992 |
| Hip Scour test L | Peak Hip Adduction Strength L | 23 | 14 | 0.995 | 0.851-1.164 | 0.952 | 0.992 |
| Hip Scour test L | Add/Abd Ratio R | 23 | 14 | 3.037 | 0.287-32.177 | 0.356 | 0.711 |
| Hip Scour test L | Add/Abd Ratio L | 23 | 14 | 6.551 | 0.592-72.485 | 0.125 | 0.687 |
| Hip Scour test L | Peak Hip External Rotation Strength R | 23 | 14 | 1.089 | 0.930-1.274 | 0.290 | 0.711 |
| Hip Scour test L | Peak Hip External Rotation Strength L | 23 | 14 | 1.066 | 0.897-1.268 | 0.468 | 0.711 |
| Hip Scour test L | Peak Hip Internal Rotation Strength R | 23 | 14 | 0.877 | 0.747-1.029 | 0.108 | 0.687 |
| Hip Scour test L | Peak Hip Internal Rotation Strength L | 23 | 14 | 0.957 | 0.849-1.079 | 0.475 | 0.711 |
| Hip Scour test L | IR/ER Ratio R | 23 | 14 | 0.019 | 0.001-0.454 | 0.014 | 0.394 |
| Hip Scour test L | IR/ER Ratio L | 23 | 14 | 0.297 | 0.039-2.229 | 0.238 | 0.711 |
| Hip Scour test L | Peak Hip Flexion Strength R | 23 | 14 | 0.981 | 0.811-1.188 | 0.846 | 0.970 |
| Hip Scour test L | Peak Hip Flexion Strength L | 23 | 14 | 1.058 | 0.896-1.249 | 0.507 | 0.711 |
| Hip Scour test L | mSEBT_A R | 23 | 14 | 1.034 | 0.977-1.094 | 0.248 | 0.711 |
| Hip Scour test L | mSEBT_A L | 23 | 14 | 0.949 | 0.856-1.051 | 0.316 | 0.711 |
| Hip Scour test L | mSEBT_PM R | 23 | 14 | 0.993 | 0.939-1.050 | 0.808 | 0.970 |
| Hip Scour test L | mSEBT_PM L | 23 | 14 | 1.000 | 0.975-1.025 | 0.979 | 0.992 |
| Hip Scour test L | mSEBT_PL R | 23 | 14 | 1.020 | 0.991-1.050 | 0.169 | 0.687 |
| Hip Scour test L | mSEBT_PL L | 23 | 14 | 1.008 | 0.920-1.104 | 0.866 | 0.970 |
| Hip Scour test L | Cross-Over Hop R | 23 | 14 | 0.994 | 0.983-1.006 | 0.337 | 0.711 |
| Hip Scour test L | Cross-Over Hop L | 23 | 14 | 0.995 | 0.982-1.007 | 0.412 | 0.711 |
| **FADIR test R** | Peak Knee Flexion Strength R | 19 | 18 | 0.957 | 0.865-1.058 | 0.389 | 0.759 |
| FADIR test R | Peak Knee Flexion Strength L | 19 | 18 | 0.935 | 0.810-1.079 | 0.360 | 0.759 |
| FADIR test R | Peak Knee Extension Strength R | 19 | 18 | 1.002 | 0.948-1.059 | 0.951 | 0.986 |
| FADIR test R | Peak Knee Extension Strength L | 19 | 18 | 1.015 | 0.947-1.087 | 0.677 | 0.845 |
| FADIR test R | F/E Ratio R | 19 | 18 | 0.207 | 0.004-11.501 | 0.442 | 0.759 |
| FADIR test R | F/E Ratio L | 19 | 18 | 0.052 | 0.001-5.190 | 0.208 | 0.729 |
| FADIR test R | Peak Hip Abduction Strength R | 19 | 18 | 0.886 | 0.696-1.128 | 0.326 | 0.759 |
| FADIR test R | Peak Hip Abduction Strength L | 19 | 18 | 0.889 | 0.680-1.163 | 0.392 | 0.759 |
| FADIR test R | Peak Hip Adduction Strength R | 19 | 18 | 0.964 | 0.832-1.117 | 0.626 | 0.835 |
| FADIR test R | Peak Hip Adduction Strength L | 19 | 18 | 0.845 | 0.708-1.007 | 0.060 | 0.729 |
| FADIR test R | Add/Abd Ratio R | 19 | 18 | 0.988 | 0.122-7.991 | 0.991 | 0.991 |
| FADIR test R | Add/Abd Ratio L | 19 | 18 | 0.208 | 0.024-1.834 | 0.158 | 0.729 |
| FADIR test R | Peak Hip External Rotation Strength R | 19 | 18 | 0.895 | 0.770-1.040 | 0.147 | 0.729 |
| FADIR test R | Peak Hip External Rotation Strength L | 19 | 18 | 0.916 | 0.781-1.074 | 0.279 | 0.759 |
| FADIR test R | Peak Hip Internal Rotation Strength R | 19 | 18 | 0.953 | 0.823-1.103 | 0.515 | 0.801 |
| FADIR test R | Peak Hip Internal Rotation Strength L | 19 | 18 | 0.984 | 0.876-1.105 | 0.787 | 0.918 |
| FADIR test R | IR/ER Ratio R | 19 | 18 | 2.250 | 0.261-19.407 | 0.461 | 0.759 |
| FADIR test R | IR/ER Ratio L | 19 | 18 | 2.211 | 0.328-14.924 | 0.415 | 0.759 |
| FADIR test R | Peak Hip Flexion Strength R | 19 | 18 | 1.037 | 0.864-1.246 | 0.694 | 0.845 |
| FADIR test R | Peak Hip Flexion Strength L | 19 | 18 | 1.048 | 0.892-1.232 | 0.568 | 0.835 |
| FADIR test R | mSEBT_A R | 19 | 18 | 1.044 | 0.976-1.117 | 0.206 | 0.729 |
| FADIR test R | mSEBT_A L | 19 | 18 | 0.966 | 0.891-1.046 | 0.393 | 0.759 |
| FADIR test R | mSEBT_PM R | 19 | 18 | 1.041 | 0.982-1.104 | 0.173 | 0.729 |
| FADIR test R | mSEBT_PM L | 19 | 18 | 1.007 | 0.982-1.032 | 0.605 | 0.835 |
| FADIR test R | mSEBT_PL R | 19 | 18 | 0.998 | 0.972-1.025 | 0.886 | 0.954 |
| FADIR test R | mSEBT_PL L | 19 | 18 | 1.008 | 0.923-1.101 | 0.856 | 0.954 |
| FADIR test R | Cross-Over Hop R | 19 | 18 | 0.992 | 0.979-1.004 | 0.187 | 0.729 |
| FADIR test R | Cross-Over Hop L | 19 | 18 | 0.990 | 0.975-1.005 | 0.174 | 0.729 |
| **FADIR test L** | Peak Knee Flexion Strength R | 22 | 15 | 0.978 | 0.885-1.080 | 0.660 | 0.799 |
| FADIR test L | Peak Knee Flexion Strength L | 22 | 15 | 0.935 | 0.811-1.077 | 0.352 | 0.799 |
| FADIR test L | Peak Knee Extension Strength R | 22 | 15 | 0.990 | 0.937-1.046 | 0.722 | 0.799 |
| FADIR test L | Peak Knee Extension Strength L | 22 | 15 | 0.974 | 0.908-1.044 | 0.460 | 0.799 |
| FADIR test L | F/E Ratio R | 22 | 15 | 0.436 | 0.009-21.449 | 0.677 | 0.799 |
| FADIR test L | F/E Ratio L | 22 | 15 | 0.473 | 0.006-35.343 | 0.734 | 0.799 |
| FADIR test L | Peak Hip Abduction Strength R | 22 | 15 | 0.960 | 0.757-1.218 | 0.736 | 0.799 |
| FADIR test L | Peak Hip Abduction Strength L | 22 | 15 | 0.764 | 0.557-1.048 | 0.095 | 0.531 |
| FADIR test L | Peak Hip Adduction Strength R | 22 | 15 | 1.045 | 0.899-1.215 | 0.568 | 0.799 |
| FADIR test L | Peak Hip Adduction Strength L | 22 | 15 | 1.043 | 0.892-1.221 | 0.597 | 0.799 |
| FADIR test L | Add/Abd Ratio R | 22 | 15 | 2.171 | 0.234-20.147 | 0.495 | 0.799 |
| FADIR test L | Add/Abd Ratio L | 22 | 15 | 3.906 | 0.429-35.572 | 0.227 | 0.794 |
| FADIR test L | Peak Hip External Rotation Strength R | 22 | 15 | 1.181 | 0.984-1.417 | 0.074 | 0.531 |
| FADIR test L | Peak Hip External Rotation Strength L | 22 | 15 | 1.172 | 0.946-1.451 | 0.147 | 0.685 |
| FADIR test L | Peak Hip Internal Rotation Strength R | 22 | 15 | 0.967 | 0.838-1.115 | 0.645 | 0.799 |
| FADIR test L | Peak Hip Internal Rotation Strength L | 22 | 15 | 1.031 | 0.916-1.160 | 0.617 | 0.799 |
| FADIR test L | IR/ER Ratio R | 22 | 15 | 0.031 | 0.002-0.588 | 0.021 | 0.531 |
| FADIR test L | IR/ER Ratio L | 22 | 15 | 0.490 | 0.074-3.255 | 0.460 | 0.799 |
| FADIR test L | Peak Hip Flexion Strength R | 22 | 15 | 0.969 | 0.803-1.170 | 0.742 | 0.799 |
| FADIR test L | Peak Hip Flexion Strength L | 22 | 15 | 1.171 | 0.974-1.407 | 0.093 | 0.531 |
| FADIR test L | mSEBT_A R | 22 | 15 | 1.041 | 0.976-1.111 | 0.224 | 0.794 |
| FADIR test L | mSEBT_A L | 22 | 15 | 0.978 | 0.908-1.054 | 0.566 | 0.799 |
| FADIR test L | mSEBT_PM R | 22 | 15 | 1.015 | 0.960-1.073 | 0.607 | 0.799 |
| FADIR test L | mSEBT_PM L | 22 | 15 | 1.001 | 0.976-1.026 | 0.945 | 0.950 |
| FADIR test L | mSEBT_PL R | 22 | 15 | 1.073 | 0.989-1.164 | 0.090 | 0.531 |
| FADIR test L | mSEBT_PL L | 22 | 15 | 1.003 | 0.917-1.097 | 0.950 | 0.950 |
| FADIR test L | Cross-Over Hop R | 22 | 15 | 0.993 | 0.982-1.005 | 0.259 | 0.799 |
| FADIR test L | Cross-Over Hop L | 22 | 15 | 0.993 | 0.981-1.006 | 0.305 | 0.799 |
| **FABER test R** | Peak Knee Flexion Strength R | 21 | 16 | 0.929 | 0.833-1.036 | 0.186 | 0.372 |
| FABER test R | Peak Knee Flexion Strength L | 21 | 16 | 0.877 | 0.751-1.024 | 0.097 | 0.372 |
| FABER test R | Peak Knee Extension Strength R | 21 | 16 | 0.990 | 0.936-1.047 | 0.723 | 0.834 |
| FABER test R | Peak Knee Extension Strength L | 21 | 16 | 0.996 | 0.928-1.068 | 0.900 | 0.933 |
| FABER test R | F/E Ratio R | 21 | 16 | 0.124 | 0.002-7.838 | 0.324 | 0.477 |
| FABER test R | F/E Ratio L | 21 | 16 | 0.042 | 0.000-4.237 | 0.178 | 0.372 |
| FABER test R | Peak Hip Abduction Strength R | 21 | 16 | 0.680 | 0.482-0.959 | 0.028 | 0.372 |
| FABER test R | Peak Hip Abduction Strength L | 21 | 16 | 0.802 | 0.594-1.083 | 0.150 | 0.372 |
| FABER test R | Peak Hip Adduction Strength R | 21 | 16 | 0.854 | 0.721-1.011 | 0.067 | 0.372 |
| FABER test R | Peak Hip Adduction Strength L | 21 | 16 | 0.782 | 0.640-0.956 | 0.016 | 0.372 |
| FABER test R | Add/Abd Ratio R | 21 | 16 | 0.647 | 0.080-5.233 | 0.683 | 0.832 |
| FABER test R | Add/Abd Ratio L | 21 | 16 | 0.270 | 0.032-2.275 | 0.229 | 0.400 |
| FABER test R | Peak Hip External Rotation Strength R | 21 | 16 | 0.881 | 0.754-1.031 | 0.114 | 0.372 |
| FABER test R | Peak Hip External Rotation Strength L | 21 | 16 | 0.879 | 0.741-1.043 | 0.139 | 0.372 |
| FABER test R | Peak Hip Internal Rotation Strength R | 21 | 16 | 0.871 | 0.734-1.033 | 0.112 | 0.372 |
| FABER test R | Peak Hip Internal Rotation Strength L | 21 | 16 | 0.934 | 0.826-1.058 | 0.283 | 0.456 |
| FABER test R | IR/ER Ratio R | 21 | 16 | 0.722 | 0.083-6.296 | 0.768 | 0.834 |
| FABER test R | IR/ER Ratio L | 21 | 16 | 1.640 | 0.240-11.219 | 0.614 | 0.781 |
| FABER test R | Peak Hip Flexion Strength R | 21 | 16 | 0.857 | 0.698-1.052 | 0.140 | 0.372 |
| FABER test R | Peak Hip Flexion Strength L | 21 | 16 | 0.915 | 0.776-1.079 | 0.293 | 0.456 |
| FABER test R | mSEBT_A R | 21 | 16 | 1.044 | 0.976-1.117 | 0.210 | 0.391 |
| FABER test R | mSEBT_A L | 21 | 16 | 0.960 | 0.881-1.045 | 0.347 | 0.486 |
| FABER test R | mSEBT_PM R | 21 | 16 | 0.998 | 0.941-1.059 | 0.956 | 0.956 |
| FABER test R | mSEBT_PM L | 21 | 16 | 0.969 | 0.928-1.013 | 0.164 | 0.372 |
| FABER test R | mSEBT_PL R | 21 | 16 | 0.996 | 0.969-1.024 | 0.775 | 0.834 |
| FABER test R | mSEBT_PL L | 21 | 16 | 0.962 | 0.877-1.056 | 0.419 | 0.558 |
| FABER test R | Cross-Over Hop R | 21 | 16 | 0.991 | 0.978-1.004 | 0.157 | 0.372 |
| FABER test R | Cross-Over Hop L | 21 | 16 | 0.988 | 0.974-1.003 | 0.114 | 0.372 |
| **FABER test L** | Peak Knee Flexion Strength R | 26 | 11 | 1.063 | 0.929-1.217 | 0.375 | 0.974 |
| FABER test L | Peak Knee Flexion Strength L | 26 | 11 | 0.967 | 0.832-1.123 | 0.656 | 0.974 |
| FABER test L | Peak Knee Extension Strength R | 26 | 11 | 0.996 | 0.939-1.057 | 0.902 | 0.974 |
| FABER test L | Peak Knee Extension Strength L | 26 | 11 | 1.001 | 0.929-1.080 | 0.970 | 0.974 |
| FABER test L | F/E Ratio R | 26 | 11 | 1.832 | 0.024-141.457 | 0.785 | 0.974 |
| FABER test L | F/E Ratio L | 26 | 11 | 0.265 | 0.002-30.467 | 0.583 | 0.974 |
| FABER test L | Peak Hip Abduction Strength R | 26 | 11 | 1.004 | 0.771-1.309 | 0.974 | 0.974 |
| FABER test L | Peak Hip Abduction Strength L | 26 | 11 | 0.927 | 0.697-1.233 | 0.602 | 0.974 |
| FABER test L | Peak Hip Adduction Strength R | 26 | 11 | 1.027 | 0.870-1.212 | 0.754 | 0.974 |
| FABER test L | Peak Hip Adduction Strength L | 26 | 11 | 1.035 | 0.871-1.230 | 0.694 | 0.974 |
| FABER test L | Add/Abd Ratio R | 26 | 11 | 1.900 | 0.141-25.586 | 0.628 | 0.974 |
| FABER test L | Add/Abd Ratio L | 26 | 11 | 4.212 | 0.321-55.302 | 0.274 | 0.974 |
| FABER test L | Peak Hip External Rotation Strength R | 26 | 11 | 1.178 | 0.945-1.468 | 0.144 | 0.974 |
| FABER test L | Peak Hip External Rotation Strength L | 26 | 11 | 1.129 | 0.893-1.428 | 0.311 | 0.974 |
| FABER test L | Peak Hip Internal Rotation Strength R | 26 | 11 | 1.009 | 0.856-1.188 | 0.916 | 0.974 |
| FABER test L | Peak Hip Internal Rotation Strength L | 26 | 11 | 1.032 | 0.900-1.184 | 0.649 | 0.974 |
| FABER test L | IR/ER Ratio R | 26 | 11 | 0.292 | 0.028-3.078 | 0.306 | 0.974 |
| FABER test L | IR/ER Ratio L | 26 | 11 | 0.871 | 0.113-6.715 | 0.895 | 0.974 |
| FABER test L | Peak Hip Flexion Strength R | 26 | 11 | 0.969 | 0.787-1.193 | 0.765 | 0.974 |
| FABER test L | Peak Hip Flexion Strength L | 26 | 11 | 1.070 | 0.895-1.279 | 0.460 | 0.974 |
| FABER test L | mSEBT_A R | 26 | 11 | 0.994 | 0.950-1.041 | 0.812 | 0.974 |
| FABER test L | mSEBT_A L | 26 | 11 | 1.042 | 0.974-1.115 | 0.233 | 0.974 |
| FABER test L | mSEBT_PM R | 26 | 11 | 0.943 | 0.869-1.024 | 0.162 | 0.974 |
| FABER test L | mSEBT_PM L | 26 | 11 | 1.003 | 0.974-1.033 | 0.828 | 0.974 |
| FABER test L | mSEBT_PL R | 26 | 11 | 0.966 | 0.907-1.028 | 0.273 | 0.974 |
| FABER test L | mSEBT_PL L | 26 | 11 | 0.979 | 0.885-1.082 | 0.676 | 0.974 |
| FABER test L | Cross-Over Hop R | 26 | 11 | 0.997 | 0.985-1.009 | 0.612 | 0.974 |
| FABER test L | Cross-Over Hop L | 26 | 11 | 0.996 | 0.983-1.009 | 0.558 | 0.974 |
| **Squeeze at 90°** | Peak Knee Flexion Strength R | 9 | 28 | 0.979 | 0.872-1.099 | 0.721 | 0.884 |
| Squeeze at 90° | Peak Knee Flexion Strength L | 9 | 28 | 0.882 | 0.729-1.069 | 0.201 | 0.664 |
| Squeeze at 90° | Peak Knee Extension Strength R | 9 | 28 | 0.961 | 0.895-1.031 | 0.269 | 0.664 |
| Squeeze at 90° | Peak Knee Extension Strength L | 9 | 28 | 0.990 | 0.910-1.078 | 0.824 | 0.884 |
| Squeeze at 90° | F/E Ratio R | 9 | 28 | 2.021 | 0.024-166.875 | 0.755 | 0.884 |
| Squeeze at 90° | F/E Ratio L | 9 | 28 | 0.041 | 0.000-13.910 | 0.282 | 0.664 |
| Squeeze at 90° | Peak Hip Abduction Strength R | 9 | 28 | 0.849 | 0.628-1.146 | 0.285 | 0.664 |
| Squeeze at 90° | Peak Hip Abduction Strength L | 9 | 28 | 0.708 | 0.478-1.049 | 0.085 | 0.664 |
| Squeeze at 90° | Peak Hip Adduction Strength R | 9 | 28 | 0.873 | 0.709-1.076 | 0.203 | 0.664 |
| Squeeze at 90° | Peak Hip Adduction Strength L | 9 | 28 | 0.949 | 0.787-1.144 | 0.583 | 0.859 |
| Squeeze at 90° | Add/Abd Ratio R | 9 | 28 | 0.612 | 0.056-6.718 | 0.688 | 0.884 |
| Squeeze at 90° | Add/Abd Ratio L | 9 | 28 | 3.312 | 0.305-35.929 | 0.325 | 0.664 |
| Squeeze at 90° | Peak Hip External Rotation Strength R | 9 | 28 | 0.917 | 0.764-1.102 | 0.356 | 0.664 |
| Squeeze at 90° | Peak Hip External Rotation Strength L | 9 | 28 | 0.838 | 0.645-1.090 | 0.189 | 0.664 |
| Squeeze at 90° | Peak Hip Internal Rotation Strength R | 9 | 28 | 0.915 | 0.758-1.104 | 0.352 | 0.664 |
| Squeeze at 90° | Peak Hip Internal Rotation Strength L | 9 | 28 | 0.871 | 0.734-1.034 | 0.114 | 0.664 |
| Squeeze at 90° | IR/ER Ratio R | 9 | 28 | 1.128 | 0.088-14.385 | 0.926 | 0.926 |
| Squeeze at 90° | IR/ER Ratio L | 9 | 28 | 0.772 | 0.091-6.527 | 0.812 | 0.884 |
| Squeeze at 90° | Peak Hip Flexion Strength R | 9 | 28 | 0.753 | 0.580-0.977 | 0.033 | 0.664 |
| Squeeze at 90° | Peak Hip Flexion Strength L | 9 | 28 | 0.773 | 0.591-1.012 | 0.061 | 0.664 |
| Squeeze at 90° | mSEBT_A R | 9 | 28 | 1.019 | 0.945-1.098 | 0.630 | 0.882 |
| Squeeze at 90° | mSEBT_A L | 9 | 28 | 1.035 | 0.946-1.133 | 0.452 | 0.704 |
| Squeeze at 90° | mSEBT_PM R | 9 | 28 | 0.993 | 0.927-1.064 | 0.853 | 0.884 |
| Squeeze at 90° | mSEBT_PM L | 9 | 28 | 1.018 | 0.974-1.063 | 0.432 | 0.704 |
| Squeeze at 90° | mSEBT_PL R | 9 | 28 | 0.997 | 0.967-1.028 | 0.853 | 0.884 |
| Squeeze at 90° | mSEBT_PL L | 9 | 28 | 0.918 | 0.800-1.054 | 0.226 | 0.664 |
| Squeeze at 90° | Cross-Over Hop R | 9 | 28 | 0.987 | 0.970-1.005 | 0.159 | 0.664 |
| Squeeze at 90° | Cross-Over Hop L | 9 | 28 | 0.992 | 0.973-1.012 | 0.416 | 0.704 |
| **Squeeze at 45°** | Peak Knee Flexion Strength R | 8 | 29 | 1.000 | 0.888-1.126 | 0.995 | 0.995 |
| Squeeze at 45° | Peak Knee Flexion Strength L | 8 | 29 | 0.885 | 0.726-1.079 | 0.227 | 0.583 |
| Squeeze at 45° | Peak Knee Extension Strength R | 8 | 29 | 0.989 | 0.925-1.058 | 0.755 | 0.990 |
| Squeeze at 45° | Peak Knee Extension Strength L | 8 | 29 | 0.998 | 0.919-1.083 | 0.955 | 0.990 |
| Squeeze at 45° | F/E Ratio R | 8 | 29 | 0.871 | 0.009-88.480 | 0.953 | 0.990 |
| Squeeze at 45° | F/E Ratio L | 8 | 29 | 0.160 | 0.001-38.182 | 0.511 | 0.795 |
| Squeeze at 45° | Peak Hip Abduction Strength R | 8 | 29 | 0.881 | 0.652-1.190 | 0.410 | 0.717 |
| Squeeze at 45° | Peak Hip Abduction Strength L | 8 | 29 | 0.805 | 0.566-1.145 | 0.227 | 0.583 |
| Squeeze at 45° | Peak Hip Adduction Strength R | 8 | 29 | 0.713 | 0.537-0.946 | 0.019 | 0.270 |
| Squeeze at 45° | Peak Hip Adduction Strength L | 8 | 29 | 0.819 | 0.642-1.045 | 0.109 | 0.507 |
| Squeeze at 45° | Add/Abd Ratio R | 8 | 29 | 0.038 | 0.001-1.667 | 0.090 | 0.504 |
| Squeeze at 45° | Add/Abd Ratio L | 8 | 29 | 0.769 | 0.058-10.258 | 0.842 | 0.990 |
| Squeeze at 45° | Peak Hip External Rotation Strength R | 8 | 29 | 0.665 | 0.452-0.978 | 0.038 | 0.355 |
| Squeeze at 45° | Peak Hip External Rotation Strength L | 8 | 29 | 0.529 | 0.316-0.885 | 0.015 | 0.270 |
| Squeeze at 45° | Peak Hip Internal Rotation Strength R | 8 | 29 | 0.882 | 0.705-1.103 | 0.271 | 0.583 |
| Squeeze at 45° | Peak Hip Internal Rotation Strength L | 8 | 29 | 0.903 | 0.760-1.071 | 0.241 | 0.583 |
| Squeeze at 45° | IR/ER Ratio R | 8 | 29 | 6.388 | 0.535-76.308 | 0.143 | 0.571 |
| Squeeze at 45° | IR/ER Ratio L | 8 | 29 | 3.738 | 0.395-35.388 | 0.250 | 0.583 |
| Squeeze at 45° | Peak Hip Flexion Strength R | 8 | 29 | 0.908 | 0.733-1.126 | 0.380 | 0.709 |
| Squeeze at 45° | Peak Hip Flexion Strength L | 8 | 29 | 0.801 | 0.633-1.014 | 0.066 | 0.459 |
| Squeeze at 45° | mSEBT_A R | 8 | 29 | 1.104 | 0.960-1.268 | 0.164 | 0.575 |
| Squeeze at 45° | mSEBT_A L | 8 | 29 | 1.031 | 0.933-1.139 | 0.553 | 0.815 |
| Squeeze at 45° | mSEBT_PM R | 8 | 29 | 1.003 | 0.940-1.071 | 0.918 | 0.990 |
| Squeeze at 45° | mSEBT_PM L | 8 | 29 | 0.996 | 0.967-1.026 | 0.786 | 0.990 |
| Squeeze at 45° | mSEBT_PL R | 8 | 29 | 1.030 | 0.967-1.098 | 0.360 | 0.709 |
| Squeeze at 45° | mSEBT_PL L | 8 | 29 | 0.994 | 0.900-1.099 | 0.907 | 0.990 |
| Squeeze at 45° | Cross-Over Hop R | 8 | 29 | 0.995 | 0.981-1.009 | 0.455 | 0.750 |
| Squeeze at 45° | Cross-Over Hop L | 8 | 29 | 0.996 | 0.981-1.011 | 0.602 | 0.843 |
| **Squeeze at 0°** | Peak Knee Flexion Strength R | 11 | 26 | 1.041 | 0.939-1.155 | 0.442 | 0.649 |
| Squeeze at 0° | Peak Knee Flexion Strength L | 11 | 26 | 1.006 | 0.865-1.170 | 0.934 | 0.934 |
| Squeeze at 0° | Peak Knee Extension Strength R | 11 | 26 | 0.970 | 0.912-1.031 | 0.326 | 0.649 |
| Squeeze at 0° | Peak Knee Extension Strength L | 11 | 26 | 0.963 | 0.891-1.042 | 0.350 | 0.649 |
| Squeeze at 0° | F/E Ratio R | 11 | 26 | 11.544 | 0.174-765.768 | 0.253 | 0.649 |
| Squeeze at 0° | F/E Ratio L | 11 | 26 | 16.210 | 0.142-1845.109 | 0.249 | 0.649 |
| Squeeze at 0° | Peak Hip Abduction Strength R | 11 | 26 | 0.903 | 0.693-1.177 | 0.450 | 0.649 |
| Squeeze at 0° | Peak Hip Abduction Strength L | 11 | 26 | 0.792 | 0.564-1.113 | 0.179 | 0.649 |
| Squeeze at 0° | Peak Hip Adduction Strength R | 11 | 26 | 0.790 | 0.616-1.012 | 0.062 | 0.649 |
| Squeeze at 0° | Peak Hip Adduction Strength L | 11 | 26 | 0.938 | 0.789-1.116 | 0.469 | 0.649 |
| Squeeze at 0° | Add/Abd Ratio R | 11 | 26 | 0.181 | 0.010-3.256 | 0.246 | 0.649 |
| Squeeze at 0° | Add/Abd Ratio L | 11 | 26 | 1.749 | 0.200-15.323 | 0.614 | 0.661 |
| Squeeze at 0° | Peak Hip External Rotation Strength R | 11 | 26 | 0.945 | 0.799-1.118 | 0.510 | 0.649 |
| Squeeze at 0° | Peak Hip External Rotation Strength L | 11 | 26 | 0.928 | 0.767-1.122 | 0.439 | 0.649 |
| Squeeze at 0° | Peak Hip Internal Rotation Strength R | 11 | 26 | 1.010 | 0.869-1.173 | 0.899 | 0.932 |
| Squeeze at 0° | Peak Hip Internal Rotation Strength L | 11 | 26 | 1.045 | 0.921-1.185 | 0.498 | 0.649 |
| Squeeze at 0° | IR/ER Ratio R | 11 | 26 | 2.844 | 0.279-29.034 | 0.378 | 0.649 |
| Squeeze at 0° | IR/ER Ratio L | 11 | 26 | 5.490 | 0.621-48.505 | 0.126 | 0.649 |
| Squeeze at 0° | Peak Hip Flexion Strength R | 11 | 26 | 1.073 | 0.874-1.318 | 0.499 | 0.649 |
| Squeeze at 0° | Peak Hip Flexion Strength L | 11 | 26 | 0.953 | 0.796-1.139 | 0.595 | 0.661 |
| Squeeze at 0° | mSEBT_A R | 11 | 26 | 1.136 | 0.981-1.316 | 0.089 | 0.649 |
| Squeeze at 0° | mSEBT_A L | 11 | 26 | 1.039 | 0.949-1.136 | 0.409 | 0.649 |
| Squeeze at 0° | mSEBT_PM R | 11 | 26 | 0.982 | 0.919-1.050 | 0.593 | 0.661 |
| Squeeze at 0° | mSEBT_PM L | 11 | 26 | 0.991 | 0.960-1.023 | 0.570 | 0.661 |
| Squeeze at 0° | mSEBT_PL R | 11 | 26 | 1.023 | 0.973-1.076 | 0.375 | 0.649 |
| Squeeze at 0° | mSEBT_PL L | 11 | 26 | 0.948 | 0.846-1.062 | 0.360 | 0.649 |
| Squeeze at 0° | Cross-Over Hop R | 11 | 26 | 0.984 | 0.968-1.001 | 0.065 | 0.649 |
| Squeeze at 0° | Cross-Over Hop L | 11 | 26 | 0.988 | 0.970-1.007 | 0.223 | 0.649 |
| **Palpation - adductor region R** | Peak Knee Flexion Strength R | 24 | 13 | 0.966 | 0.874-1.067 | 0.497 | 0.663 |
| Palpation - adductor region R | Peak Knee Flexion Strength L | 24 | 13 | 0.901 | 0.779-1.043 | 0.164 | 0.489 |
| Palpation - adductor region R | Peak Knee Extension Strength R | 24 | 13 | 0.938 | 0.880-1.001 | 0.052 | 0.488 |
| Palpation - adductor region R | Peak Knee Extension Strength L | 24 | 13 | 0.908 | 0.831-0.992 | 0.032 | 0.451 |
| Palpation - adductor region R | F/E Ratio R | 24 | 13 | 19.905 | 0.251-1579.617 | 0.180 | 0.489 |
| Palpation - adductor region R | F/E Ratio L | 24 | 13 | 13.906 | 0.114-1693.245 | 0.283 | 0.549 |
| Palpation - adductor region R | Peak Hip Abduction Strength R | 24 | 13 | 0.837 | 0.640-1.094 | 0.192 | 0.489 |
| Palpation - adductor region R | Peak Hip Abduction Strength L | 24 | 13 | 0.897 | 0.686-1.173 | 0.428 | 0.631 |
| Palpation - adductor region R | Peak Hip Adduction Strength R | 24 | 13 | 0.896 | 0.763-1.052 | 0.180 | 0.489 |
| Palpation - adductor region R | Peak Hip Adduction Strength L | 24 | 13 | 0.815 | 0.677-0.982 | 0.032 | 0.451 |
| Palpation - adductor region R | Add/Abd Ratio R | 24 | 13 | 0.572 | 0.069-4.761 | 0.605 | 0.706 |
| Palpation - adductor region R | Add/Abd Ratio L | 24 | 13 | 0.193 | 0.022-1.711 | 0.140 | 0.489 |
| Palpation - adductor region R | Peak Hip External Rotation Strength R | 24 | 13 | 0.865 | 0.737-1.016 | 0.077 | 0.489 |
| Palpation - adductor region R | Peak Hip External Rotation Strength L | 24 | 13 | 0.875 | 0.736-1.041 | 0.132 | 0.489 |
| Palpation - adductor region R | Peak Hip Internal Rotation Strength R | 24 | 13 | 0.909 | 0.780-1.060 | 0.223 | 0.518 |
| Palpation - adductor region R | Peak Hip Internal Rotation Strength L | 24 | 13 | 0.938 | 0.830-1.061 | 0.308 | 0.549 |
| Palpation - adductor region R | IR/ER Ratio R | 24 | 13 | 1.560 | 0.166-14.670 | 0.697 | 0.751 |
| Palpation - adductor region R | IR/ER Ratio L | 24 | 13 | 1.736 | 0.245-12.322 | 0.581 | 0.706 |
| Palpation - adductor region R | Peak Hip Flexion Strength R | 24 | 13 | 0.976 | 0.810-1.177 | 0.803 | 0.833 |
| Palpation - adductor region R | Peak Hip Flexion Strength L | 24 | 13 | 0.881 | 0.741-1.048 | 0.152 | 0.489 |
| Palpation - adductor region R | mSEBT_A R | 24 | 13 | 1.019 | 0.973-1.067 | 0.417 | 0.631 |
| Palpation - adductor region R | mSEBT_A L | 24 | 13 | 1.024 | 0.960-1.092 | 0.476 | 0.663 |
| Palpation - adductor region R | mSEBT_PM R | 24 | 13 | 0.997 | 0.937-1.060 | 0.920 | 0.920 |
| Palpation - adductor region R | mSEBT_PM L | 24 | 13 | 0.982 | 0.947-1.019 | 0.333 | 0.549 |
| Palpation - adductor region R | mSEBT_PL R | 24 | 13 | 0.979 | 0.939-1.021 | 0.322 | 0.549 |
| Palpation - adductor region R | mSEBT_PL L | 24 | 13 | 0.981 | 0.894-1.077 | 0.692 | 0.751 |
| Palpation - adductor region R | Cross-Over Hop R | 24 | 13 | 0.996 | 0.984-1.009 | 0.570 | 0.706 |
| Palpation - adductor region R | Cross-Over Hop L | 24 | 13 | 0.992 | 0.978-1.006 | 0.240 | 0.518 |
| **Palpation - adductor region L** | Peak Knee Flexion Strength R | 26 | 9 | 0.992 | 0.891-1.106 | 0.891 | 0.924 |
| Palpation - adductor region L | Peak Knee Flexion Strength L | 26 | 9 | 0.968 | 0.830-1.129 | 0.681 | 0.877 |
| Palpation - adductor region L | Peak Knee Extension Strength R | 26 | 9 | 0.965 | 0.902-1.032 | 0.295 | 0.877 |
| Palpation - adductor region L | Peak Knee Extension Strength L | 26 | 9 | 0.953 | 0.877-1.036 | 0.260 | 0.877 |
| Palpation - adductor region L | F/E Ratio R | 26 | 9 | 5.093 | 0.052-501.929 | 0.487 | 0.877 |
| Palpation - adductor region L | F/E Ratio L | 26 | 9 | 6.671 | 0.030-1484.596 | 0.491 | 0.877 |
| Palpation - adductor region L | Peak Hip Abduction Strength R | 26 | 9 | 0.843 | 0.632-1.123 | 0.243 | 0.877 |
| Palpation - adductor region L | Peak Hip Abduction Strength L | 26 | 9 | 0.869 | 0.651-1.160 | 0.341 | 0.877 |
| Palpation - adductor region L | Peak Hip Adduction Strength R | 26 | 9 | 0.962 | 0.816-1.134 | 0.648 | 0.877 |
| Palpation - adductor region L | Peak Hip Adduction Strength L | 26 | 9 | 0.877 | 0.727-1.059 | 0.172 | 0.877 |
| Palpation - adductor region L | Add/Abd Ratio R | 26 | 9 | 1.557 | 0.139-17.486 | 0.720 | 0.877 |
| Palpation - adductor region L | Add/Abd Ratio L | 26 | 9 | 0.631 | 0.062-6.382 | 0.697 | 0.877 |
| Palpation - adductor region L | Peak Hip External Rotation Strength R | 26 | 9 | 0.931 | 0.789-1.097 | 0.391 | 0.877 |
| Palpation - adductor region L | Peak Hip External Rotation Strength L | 26 | 9 | 0.986 | 0.831-1.169 | 0.868 | 0.924 |
| Palpation - adductor region L | Peak Hip Internal Rotation Strength R | 26 | 9 | 0.940 | 0.806-1.096 | 0.429 | 0.877 |
| Palpation - adductor region L | Peak Hip Internal Rotation Strength L | 26 | 9 | 0.935 | 0.814-1.073 | 0.336 | 0.877 |
| Palpation - adductor region L | IR/ER Ratio R | 26 | 9 | 1.623 | 0.114-23.000 | 0.721 | 0.877 |
| Palpation - adductor region L | IR/ER Ratio L | 26 | 9 | 0.563 | 0.051-6.184 | 0.639 | 0.877 |
| Palpation - adductor region L | Peak Hip Flexion Strength R | 26 | 9 | 1.185 | 0.956-1.468 | 0.121 | 0.877 |
| Palpation - adductor region L | Peak Hip Flexion Strength L | 26 | 9 | 0.941 | 0.763-1.161 | 0.573 | 0.877 |
| Palpation - adductor region L | mSEBT_A R | 26 | 9 | 1.028 | 0.977-1.081 | 0.286 | 0.877 |
| Palpation - adductor region L | mSEBT_A L | 26 | 9 | 1.061 | 0.971-1.160 | 0.188 | 0.877 |
| Palpation - adductor region L | mSEBT_PM R | 26 | 9 | 1.029 | 0.961-1.101 | 0.414 | 0.877 |
| Palpation - adductor region L | mSEBT_PM L | 26 | 9 | 0.996 | 0.964-1.028 | 0.783 | 0.914 |
| Palpation - adductor region L | mSEBT_PL R | 26 | 9 | 0.997 | 0.965-1.030 | 0.855 | 0.924 |
| Palpation - adductor region L | mSEBT_PL L | 26 | 9 | 1.148 | 0.979-1.346 | 0.089 | 0.877 |
| Palpation - adductor region L | Cross-Over Hop R | 26 | 9 | 1.000 | 0.987-1.014 | 0.964 | 0.964 |
| Palpation - adductor region L | Cross-Over Hop L | 26 | 9 | 1.003 | 0.987-1.020 | 0.691 | 0.877 |
| **Palpation - pubic symphysis** | Peak Knee Flexion Strength R | 17 | 19 | 0.971 | 0.869-1.086 | 0.605 | 0.811 |
| Palpation - pubic symphysis | Peak Knee Flexion Strength L | 17 | 19 | 0.908 | 0.772-1.067 | 0.239 | 0.803 |
| Palpation - pubic symphysis | Peak Knee Extension Strength R | 17 | 19 | 0.983 | 0.925-1.044 | 0.571 | 0.811 |
| Palpation - pubic symphysis | Peak Knee Extension Strength L | 17 | 19 | 0.927 | 0.852-1.008 | 0.076 | 0.713 |
| Palpation - pubic symphysis | F/E Ratio R | 17 | 19 | 0.829 | 0.015-47.348 | 0.928 | 0.962 |
| Palpation - pubic symphysis | F/E Ratio L | 17 | 19 | 11.650 | 0.101-1349.587 | 0.311 | 0.803 |
| Palpation - pubic symphysis | Peak Hip Abduction Strength R | 17 | 19 | 0.935 | 0.722-1.210 | 0.608 | 0.811 |
| Palpation - pubic symphysis | Peak Hip Abduction Strength L | 17 | 19 | 0.764 | 0.545-1.072 | 0.120 | 0.803 |
| Palpation - pubic symphysis | Peak Hip Adduction Strength R | 17 | 19 | 0.982 | 0.843-1.145 | 0.819 | 0.887 |
| Palpation - pubic symphysis | Peak Hip Adduction Strength L | 17 | 19 | 0.886 | 0.740-1.061 | 0.189 | 0.803 |
| Palpation - pubic symphysis | Add/Abd Ratio R | 17 | 19 | 0.996 | 0.116-8.535 | 0.997 | 0.997 |
| Palpation - pubic symphysis | Add/Abd Ratio L | 17 | 19 | 0.769 | 0.077-7.720 | 0.823 | 0.887 |
| Palpation - pubic symphysis | Peak Hip External Rotation Strength R | 17 | 19 | 1.061 | 0.907-1.240 | 0.459 | 0.803 |
| Palpation - pubic symphysis | Peak Hip External Rotation Strength L | 17 | 19 | 1.022 | 0.869-1.203 | 0.791 | 0.887 |
| Palpation - pubic symphysis | Peak Hip Internal Rotation Strength R | 17 | 19 | 0.973 | 0.842-1.126 | 0.716 | 0.871 |
| Palpation - pubic symphysis | Peak Hip Internal Rotation Strength L | 17 | 19 | 1.049 | 0.927-1.187 | 0.450 | 0.803 |
| Palpation - pubic symphysis | IR/ER Ratio R | 17 | 19 | 0.179 | 0.012-2.575 | 0.206 | 0.803 |
| Palpation - pubic symphysis | IR/ER Ratio L | 17 | 19 | 2.271 | 0.321-16.042 | 0.411 | 0.803 |
| Palpation - pubic symphysis | Peak Hip Flexion Strength R | 17 | 19 | 1.066 | 0.872-1.304 | 0.531 | 0.811 |
| Palpation - pubic symphysis | Peak Hip Flexion Strength L | 17 | 19 | 1.131 | 0.936-1.367 | 0.202 | 0.803 |
| Palpation - pubic symphysis | mSEBT_A R | 17 | 19 | 1.058 | 0.945-1.184 | 0.330 | 0.803 |
| Palpation - pubic symphysis | mSEBT_A L | 17 | 19 | 1.058 | 0.957-1.169 | 0.274 | 0.803 |
| Palpation - pubic symphysis | mSEBT_PM R | 17 | 19 | 0.981 | 0.924-1.041 | 0.530 | 0.811 |
| Palpation - pubic symphysis | mSEBT_PM L | 17 | 19 | 0.987 | 0.959-1.015 | 0.354 | 0.803 |
| Palpation - pubic symphysis | mSEBT_PL R | 17 | 19 | 1.012 | 0.982-1.044 | 0.432 | 0.803 |
| Palpation - pubic symphysis | mSEBT_PL L | 17 | 19 | 0.978 | 0.888-1.077 | 0.647 | 0.823 |
| Palpation - pubic symphysis | Cross-Over Hop R | 17 | 19 | 0.986 | 0.972-1.001 | 0.060 | 0.713 |
| Palpation - pubic symphysis | Cross-Over Hop L | 17 | 19 | 0.982 | 0.963-1.001 | 0.058 | 0.713 |
| **Palpation - quadriceps region R** | Peak Knee Flexion Strength R | 17 | 20 | 0.967 | 0.876-1.068 | 0.511 | 0.715 |
| Palpation - quadriceps region R | Peak Knee Flexion Strength L | 17 | 20 | 0.950 | 0.828-1.089 | 0.460 | 0.678 |
| Palpation - quadriceps region R | Peak Knee Extension Strength R | 17 | 20 | 1.028 | 0.972-1.086 | 0.334 | 0.584 |
| Palpation - quadriceps region R | Peak Knee Extension Strength L | 17 | 20 | 1.010 | 0.945-1.080 | 0.775 | 0.863 |
| Palpation - quadriceps region R | F/E Ratio R | 17 | 20 | 0.021 | 0.000-1.633 | 0.082 | 0.502 |
| Palpation - quadriceps region R | F/E Ratio L | 17 | 20 | 0.155 | 0.002-12.042 | 0.402 | 0.625 |
| Palpation - quadriceps region R | Peak Hip Abduction Strength R | 17 | 20 | 0.708 | 0.526-0.953 | 0.023 | 0.502 |
| Palpation - quadriceps region R | Peak Hip Abduction Strength L | 17 | 20 | 0.769 | 0.567-1.041 | 0.090 | 0.502 |
| Palpation - quadriceps region R | Peak Hip Adduction Strength R | 17 | 20 | 0.961 | 0.832-1.110 | 0.586 | 0.782 |
| Palpation - quadriceps region R | Peak Hip Adduction Strength L | 17 | 20 | 0.890 | 0.757-1.047 | 0.160 | 0.502 |
| Palpation - quadriceps region R | Add/Abd Ratio R | 17 | 20 | 3.056 | 0.355-26.314 | 0.309 | 0.577 |
| Palpation - quadriceps region R | Add/Abd Ratio L | 17 | 20 | 1.101 | 0.154-7.880 | 0.923 | 0.923 |
| Palpation - quadriceps region R | Peak Hip External Rotation Strength R | 17 | 20 | 0.982 | 0.855-1.128 | 0.798 | 0.863 |
| Palpation - quadriceps region R | Peak Hip External Rotation Strength L | 17 | 20 | 0.966 | 0.827-1.127 | 0.659 | 0.832 |
| Palpation - quadriceps region R | Peak Hip Internal Rotation Strength R | 17 | 20 | 0.872 | 0.733-1.036 | 0.119 | 0.502 |
| Palpation - quadriceps region R | Peak Hip Internal Rotation Strength L | 17 | 20 | 1.024 | 0.914-1.147 | 0.684 | 0.832 |
| Palpation - quadriceps region R | IR/ER Ratio R | 17 | 20 | 0.172 | 0.017-1.749 | 0.137 | 0.502 |
| Palpation - quadriceps region R | IR/ER Ratio L | 17 | 20 | 2.665 | 0.407-17.463 | 0.307 | 0.577 |
| Palpation - quadriceps region R | Peak Hip Flexion Strength R | 17 | 20 | 0.845 | 0.694-1.029 | 0.094 | 0.502 |
| Palpation - quadriceps region R | Peak Hip Flexion Strength L | 17 | 20 | 0.915 | 0.778-1.076 | 0.283 | 0.577 |
| Palpation - quadriceps region R | mSEBT_A R | 17 | 20 | 1.043 | 0.978-1.112 | 0.197 | 0.502 |
| Palpation - quadriceps region R | mSEBT_A L | 17 | 20 | 0.932 | 0.844-1.030 | 0.169 | 0.502 |
| Palpation - quadriceps region R | mSEBT_PM R | 17 | 20 | 0.994 | 0.943-1.048 | 0.832 | 0.863 |
| Palpation - quadriceps region R | mSEBT_PM L | 17 | 20 | 0.989 | 0.964-1.014 | 0.387 | 0.625 |
| Palpation - quadriceps region R | mSEBT_PL R | 17 | 20 | 1.003 | 0.979-1.028 | 0.812 | 0.863 |
| Palpation - quadriceps region R | mSEBT_PL L | 17 | 20 | 0.952 | 0.871-1.041 | 0.280 | 0.577 |
| Palpation - quadriceps region R | Cross-Over Hop R | 17 | 20 | 0.992 | 0.981-1.004 | 0.195 | 0.502 |
| Palpation - quadriceps region R | Cross-Over Hop L | 17 | 20 | 0.991 | 0.977-1.004 | 0.187 | 0.502 |
| **Palpation - quadriceps region L** | Peak Knee Flexion Strength R | 23 | 14 | 1.010 | 0.914-1.115 | 0.848 | 0.913 |
| Palpation - quadriceps region L | Peak Knee Flexion Strength L | 23 | 14 | 0.964 | 0.840-1.106 | 0.601 | 0.842 |
| Palpation - quadriceps region L | Peak Knee Extension Strength R | 23 | 14 | 1.019 | 0.963-1.077 | 0.513 | 0.780 |
| Palpation - quadriceps region L | Peak Knee Extension Strength L | 23 | 14 | 1.023 | 0.954-1.097 | 0.529 | 0.780 |
| Palpation - quadriceps region L | F/E Ratio R | 23 | 14 | 0.177 | 0.003-9.074 | 0.389 | 0.777 |
| Palpation - quadriceps region L | F/E Ratio L | 23 | 14 | 0.071 | 0.001-5.964 | 0.242 | 0.677 |
| Palpation - quadriceps region L | Peak Hip Abduction Strength R | 23 | 14 | 0.829 | 0.636-1.080 | 0.165 | 0.578 |
| Palpation - quadriceps region L | Peak Hip Abduction Strength L | 23 | 14 | 0.776 | 0.572-1.053 | 0.103 | 0.577 |
| Palpation - quadriceps region L | Peak Hip Adduction Strength R | 23 | 14 | 0.952 | 0.821-1.104 | 0.516 | 0.780 |
| Palpation - quadriceps region L | Peak Hip Adduction Strength L | 23 | 14 | 0.921 | 0.787-1.079 | 0.308 | 0.677 |
| Palpation - quadriceps region L | Add/Abd Ratio R | 23 | 14 | 1.345 | 0.158-11.423 | 0.786 | 0.880 |
| Palpation - quadriceps region L | Add/Abd Ratio L | 23 | 14 | 1.374 | 0.174-10.865 | 0.763 | 0.880 |
| Palpation - quadriceps region L | Peak Hip External Rotation Strength R | 23 | 14 | 0.994 | 0.862-1.147 | 0.939 | 0.973 |
| Palpation - quadriceps region L | Peak Hip External Rotation Strength L | 23 | 14 | 0.966 | 0.829-1.126 | 0.658 | 0.877 |
| Palpation - quadriceps region L | Peak Hip Internal Rotation Strength R | 23 | 14 | 0.911 | 0.782-1.061 | 0.230 | 0.677 |
| Palpation - quadriceps region L | Peak Hip Internal Rotation Strength L | 23 | 14 | 1.000 | 0.890-1.125 | 0.995 | 0.995 |
| Palpation - quadriceps region L | IR/ER Ratio R | 23 | 14 | 0.161 | 0.016-1.647 | 0.124 | 0.577 |
| Palpation - quadriceps region L | IR/ER Ratio L | 23 | 14 | 2.079 | 0.298-14.494 | 0.460 | 0.780 |
| Palpation - quadriceps region L | Peak Hip Flexion Strength R | 23 | 14 | 0.769 | 0.605-0.978 | 0.032 | 0.358 |
| Palpation - quadriceps region L | Peak Hip Flexion Strength L | 23 | 14 | 0.817 | 0.675-0.989 | 0.038 | 0.358 |
| Palpation - quadriceps region L | mSEBT_A R | 23 | 14 | 0.969 | 0.913-1.028 | 0.300 | 0.677 |
| Palpation - quadriceps region L | mSEBT_A L | 23 | 14 | 0.968 | 0.895-1.047 | 0.416 | 0.777 |
| Palpation - quadriceps region L | mSEBT_PM R | 23 | 14 | 1.008 | 0.952-1.067 | 0.780 | 0.880 |
| Palpation - quadriceps region L | mSEBT_PM L | 23 | 14 | 0.995 | 0.967-1.022 | 0.700 | 0.880 |
| Palpation - quadriceps region L | mSEBT_PL R | 23 | 14 | 0.944 | 0.871-1.023 | 0.161 | 0.578 |
| Palpation - quadriceps region L | mSEBT_PL L | 23 | 14 | 0.954 | 0.869-1.046 | 0.314 | 0.677 |
| Palpation - quadriceps region L | Cross-Over Hop R | 23 | 14 | 0.989 | 0.976-1.002 | 0.092 | 0.577 |
| Palpation - quadriceps region L | Cross-Over Hop L | 23 | 14 | 0.982 | 0.966-0.998 | 0.027 | 0.358 |
| **Palpation - lower abdominal region R** | Peak Knee Flexion Strength R | 16 | 21 | 1.042 | 0.943-1.151 | 0.422 | 0.882 |
| Palpation - lower abdominal region R | Peak Knee Flexion Strength L | 16 | 21 | 0.971 | 0.846-1.115 | 0.679 | 0.901 |
| Palpation - lower abdominal region R | Peak Knee Extension Strength R | 16 | 21 | 1.007 | 0.953-1.064 | 0.814 | 0.901 |
| Palpation - lower abdominal region R | Peak Knee Extension Strength L | 16 | 21 | 0.986 | 0.920-1.056 | 0.686 | 0.901 |
| Palpation - lower abdominal region R | F/E Ratio R | 16 | 21 | 0.994 | 0.021-47.892 | 0.998 | 0.998 |
| Palpation - lower abdominal region R | F/E Ratio L | 16 | 21 | 0.565 | 0.007-44.171 | 0.797 | 0.901 |
| Palpation - lower abdominal region R | Peak Hip Abduction Strength R | 16 | 21 | 0.915 | 0.721-1.161 | 0.465 | 0.882 |
| Palpation - lower abdominal region R | Peak Hip Abduction Strength L | 16 | 21 | 0.726 | 0.520-1.013 | 0.060 | 0.523 |
| Palpation - lower abdominal region R | Peak Hip Adduction Strength R | 16 | 21 | 0.983 | 0.851-1.137 | 0.821 | 0.901 |
| Palpation - lower abdominal region R | Peak Hip Adduction Strength L | 16 | 21 | 0.982 | 0.841-1.146 | 0.814 | 0.901 |
| Palpation - lower abdominal region R | Add/Abd Ratio R | 16 | 21 | 1.600 | 0.199-12.878 | 0.659 | 0.901 |
| Palpation - lower abdominal region R | Add/Abd Ratio L | 16 | 21 | 3.715 | 0.434-31.822 | 0.231 | 0.588 |
| Palpation - lower abdominal region R | Peak Hip External Rotation Strength R | 16 | 21 | 1.103 | 0.948-1.284 | 0.204 | 0.588 |
| Palpation - lower abdominal region R | Peak Hip External Rotation Strength L | 16 | 21 | 1.016 | 0.872-1.185 | 0.836 | 0.901 |
| Palpation - lower abdominal region R | Peak Hip Internal Rotation Strength R | 16 | 21 | 0.947 | 0.816-1.099 | 0.473 | 0.882 |
| Palpation - lower abdominal region R | Peak Hip Internal Rotation Strength L | 16 | 21 | 1.091 | 0.963-1.237 | 0.171 | 0.588 |
| Palpation - lower abdominal region R | IR/ER Ratio R | 16 | 21 | 0.018 | 0.001-0.495 | 0.018 | 0.490 |
| Palpation - lower abdominal region R | IR/ER Ratio L | 16 | 21 | 4.350 | 0.551-34.331 | 0.163 | 0.588 |
| Palpation - lower abdominal region R | Peak Hip Flexion Strength R | 16 | 21 | 0.803 | 0.649-0.994 | 0.044 | 0.523 |
| Palpation - lower abdominal region R | Peak Hip Flexion Strength L | 16 | 21 | 0.948 | 0.806-1.116 | 0.521 | 0.901 |
| Palpation - lower abdominal region R | mSEBT_A R | 16 | 21 | 0.989 | 0.946-1.034 | 0.632 | 0.901 |
| Palpation - lower abdominal region R | mSEBT_A L | 16 | 21 | 0.921 | 0.825-1.029 | 0.145 | 0.588 |
| Palpation - lower abdominal region R | mSEBT_PM R | 16 | 21 | 0.996 | 0.944-1.052 | 0.894 | 0.927 |
| Palpation - lower abdominal region R | mSEBT_PM L | 16 | 21 | 0.996 | 0.972-1.021 | 0.760 | 0.901 |
| Palpation - lower abdominal region R | mSEBT_PL R | 16 | 21 | 1.037 | 0.990-1.087 | 0.128 | 0.588 |
| Palpation - lower abdominal region R | mSEBT_PL L | 16 | 21 | 0.963 | 0.879-1.056 | 0.424 | 0.882 |
| Palpation - lower abdominal region R | Cross-Over Hop R | 16 | 21 | 0.992 | 0.980-1.005 | 0.217 | 0.588 |
| Palpation - lower abdominal region R | Cross-Over Hop L | 16 | 21 | 0.985 | 0.969-1.001 | 0.075 | 0.523 |
| **Palpation - lower abdominal region L** | Peak Knee Flexion Strength R | 22 | 15 | 1.058 | 0.941-1.189 | 0.348 | 0.811 |
| Palpation - lower abdominal region L | Peak Knee Flexion Strength L | 22 | 15 | 0.973 | 0.842-1.123 | 0.707 | 0.864 |
| Palpation - lower abdominal region L | Peak Knee Extension Strength R | 22 | 15 | 0.988 | 0.932-1.047 | 0.689 | 0.864 |
| Palpation - lower abdominal region L | Peak Knee Extension Strength L | 22 | 15 | 0.979 | 0.908-1.054 | 0.569 | 0.864 |
| Palpation - lower abdominal region L | F/E Ratio R | 22 | 15 | 8.004 | 0.113-565.835 | 0.338 | 0.811 |
| Palpation - lower abdominal region L | F/E Ratio L | 22 | 15 | 1.140 | 0.011-113.505 | 0.956 | 0.976 |
| Palpation - lower abdominal region L | Peak Hip Abduction Strength R | 22 | 15 | 0.979 | 0.761-1.260 | 0.869 | 0.974 |
| Palpation - lower abdominal region L | Peak Hip Abduction Strength L | 22 | 15 | 0.955 | 0.727-1.254 | 0.740 | 0.864 |
| Palpation - lower abdominal region L | Peak Hip Adduction Strength R | 22 | 15 | 0.887 | 0.753-1.046 | 0.155 | 0.811 |
| Palpation - lower abdominal region L | Peak Hip Adduction Strength L | 22 | 15 | 0.909 | 0.766-1.079 | 0.277 | 0.811 |
| Palpation - lower abdominal region L | Add/Abd Ratio R | 22 | 15 | 0.316 | 0.035-2.884 | 0.307 | 0.811 |
| Palpation - lower abdominal region L | Add/Abd Ratio L | 22 | 15 | 0.618 | 0.073-5.234 | 0.659 | 0.864 |
| Palpation - lower abdominal region L | Peak Hip External Rotation Strength R | 22 | 15 | 0.960 | 0.821-1.121 | 0.604 | 0.864 |
| Palpation - lower abdominal region L | Peak Hip External Rotation Strength L | 22 | 15 | 0.925 | 0.787-1.087 | 0.345 | 0.811 |
| Palpation - lower abdominal region L | Peak Hip Internal Rotation Strength R | 22 | 15 | 0.955 | 0.817-1.117 | 0.568 | 0.864 |
| Palpation - lower abdominal region L | Peak Hip Internal Rotation Strength L | 22 | 15 | 1.005 | 0.882-1.144 | 0.943 | 0.976 |
| Palpation - lower abdominal region L | IR/ER Ratio R | 22 | 15 | 0.680 | 0.071-6.505 | 0.738 | 0.864 |
| Palpation - lower abdominal region L | IR/ER Ratio L | 22 | 15 | 4.705 | 0.464-47.695 | 0.190 | 0.811 |
| Palpation - lower abdominal region L | Peak Hip Flexion Strength R | 22 | 15 | 0.959 | 0.790-1.163 | 0.667 | 0.864 |
| Palpation - lower abdominal region L | Peak Hip Flexion Strength L | 22 | 15 | 0.903 | 0.759-1.074 | 0.248 | 0.811 |
| Palpation - lower abdominal region L | mSEBT_A R | 22 | 15 | 0.968 | 0.914-1.025 | 0.260 | 0.811 |
| Palpation - lower abdominal region L | mSEBT_A L | 22 | 15 | 1.036 | 0.965-1.112 | 0.329 | 0.811 |
| Palpation - lower abdominal region L | mSEBT_PM R | 22 | 15 | 0.976 | 0.910-1.047 | 0.505 | 0.864 |
| Palpation - lower abdominal region L | mSEBT_PM L | 22 | 15 | 0.989 | 0.955-1.023 | 0.512 | 0.864 |
| Palpation - lower abdominal region L | mSEBT_PL R | 22 | 15 | 0.981 | 0.942-1.021 | 0.345 | 0.811 |
| Palpation - lower abdominal region L | mSEBT_PL L | 22 | 15 | 1.002 | 0.910-1.103 | 0.976 | 0.976 |
| Palpation - lower abdominal region L | Cross-Over Hop R | 22 | 15 | 0.998 | 0.986-1.010 | 0.737 | 0.864 |
| Palpation - lower abdominal region L | Cross-Over Hop L | 22 | 15 | 0.991 | 0.976-1.006 | 0.225 | 0.811 |

Abbreviations: OR, odds ratio; CI, confidence interval; FDR, false discovery rate; BMI, body mass index; mSEBT, modified Star Excursion Balance Test; F/E, flexion/extension; Add/Abd, adduction/abduction; IR/ER, internal/external rotation; L, left; R, right. Two observations were missing for left adductor-region palpation and one observation was missing for pubic symphysis palpation.
